# Establishing a core outcome set for creatine transporter deficiency and guanidinoacetate methyltransferase deficiency

**DOI:** 10.1186/s13023-025-03900-3

**Published:** 2025-08-07

**Authors:** Zahra Nasseri Moghaddam, Emily K. Reinhardt, Audrey Thurm, Beth K. Potter, Maureen Smith, Celeste Graham, Beth H. Tiller, Steven A. Baker, Deborah A. Bilder, Regina Bogar, Jacobus Britz, Rachel Cafferty, Daniel P. Coller, Ton J. DeGrauw, Vicky Hall, Gerald S. Lipshutz, Nicola Longo, Saadet Mercimek-Andrews, Judith S. Miller, Marzia Pasquali, Gajja S. Salomons, Andreas Schulze, Celine P. Wheaton, Kayla F. Williams, Sarah P. Young, Jasmine Li, Sofia Balog, Theresa Selucky, Sylvia Stöckler-Ipsiroglu, Heidi Wallis

**Affiliations:** 1 Department of Pediatrics, University of British Columbia, Vancouver, BC Canada; 2 Board of Directors & Staff, Association for Creatine Deficiencies, Carlsbad, CA USA; 3 National Institute of Mental Health, NIH, Bethesda, MD USA; 4 School of Epidemiology and Public Health, Faculty of Medicine, University of Ottawa, Ottawa, ON Canada; 5 Patient Partner, University of Ottawa, Ottawa, ON Canada; 6 Patient/Caregiver Partner, Association for Creatine Deficiencies, Carlsbad, CA USA; 7 Department of Pathology, University of Utah, Salt Lake City, UT USA; 8 Huntsman Mental Health Institute, University of Utah, Salt Lake City, UT USA; 9 Scientific Medical Advisory Board, Association for Creatine Deficiencies, Carlsbad, CA USA; 10 David Geffen School of Medicine, University of California-Los Angeles, Los Angeles, CA USA; 11 Department of Medical Genetics, Faculty of Medicine & Dentistry, University of Alberta, Edmonton, AB Canada; 12 Department of Child and Adolescent Psychiatry and Behavioral Sciences, Center for Autism Research, Children’s Hospital of Philadelphia, Philadelphia, PA USA; 13 ARUP Laboratories, Department of Pathology, University of Utah, Salt Lake City, UT USA; 14 Laboratory Genetic Metabolic Diseases, Amsterdam Gastroenterology Endocrinology Metabolism, Amsterdam University Medical Centers, University of Amsterdam, Amsterdam, The Netherlands; 15 Hospital for Sick Children, University of Toronto, Toronto, ON Canada; 16 Division of Genetics and Metabolism, Department of Pediatrics, Duke University Medical Center, Durham, NC USA; 17 Fine Point Consulting, Los Angeles, CA USA; 18 Division of Biochemical Genetics, British Columbia Children’s Hospital, Vancouver, BC Canada; 19 Present Address: Ultragenyx Argentina S.R.L., Buenos Aires, Argentina

**Keywords:** Core outcome set (COS), Rare disease, Creatine transporter deficiency, Guanidinoacetate methyltransferase deficiency, Patient-centered research, Patient focused drug development

## Abstract

**Background:**

Creatine transporter (CTD) and guanidinoacetate methyltransferase (GAMT) deficiencies are rare inborn errors of creatine metabolism, resulting in cerebral creatine deficiency. Patients with either condition commonly exhibit intellectual and developmental disabilities, often accompanied by behavior problems, delayed speech, seizures, and motor impairments. There is currently no efficacious treatment for CTD, while current management for GAMT requires lifelong treatment with a protein restricted diet and intake of high amounts of oral supplements. Efforts to conduct clinical trials on potential treatments for these disorders are made more difficult by the lack of clinical and patient-derived meaningful outcomes. A core outcome set (COS) can facilitate consistent use of outcomes in studies. The current effort included patient and caregiver perspectives into the outcome selection of a COS for CTD and GAMT.

**Results:**

We partnered with caregivers and health professionals to establish the first COS for CTD and GAMT. The COS developed includes seven outcomes (“Adaptive Functioning”, “Cognitive Functioning”, “Emotional Dysregulation”, “MRS Brain Creatine”, “Seizure/Convulsions”, “Expressive Communication”, and “Fine Motor Functions”) for both CTD and GAMT, and an additional outcome for GAMT (“Serum/Plasma Guanidinoacetate”) that are important to stakeholders and consequently should be considered for measurement in every clinical trial. Caregivers were valued partners throughout the COS development process, which increased community engagement and facilitated caregiver empowerment.

**Conclusions:**

Development of this COS illustrates a patient-centered approach for clinical trial readiness for CTD and GAMT that if utilized will make clinical trial results comparable, minimize bias in clinical trial outcome selection, and promote efficient use of resources.

**Supplementary Information:**

The online version contains supplementary material available at 10.1186/s13023-025-03900-3.

## Background

Creatine transporter (CTD, MIM:300352) and guanidinoacetate methyltransferase (GAMT, MIM:612736) deficiencies are rare inborn errors of creatine metabolism and transport, resulting in cerebral creatine deficiency [1–3]. CTD is an X-linked disorder caused by hemizygous or heterozygous pathogenic variants in *SLC6A8* (identified in 2001) [4], disrupting the cellular uptake of creatine in the brain [3, 5]. GAMT is an autosomal recessive disorder caused by biallelic pathogenic variants in *GAMT* (identified in 1994) [6], preventing the biosynthesis of creatine [7]. GAMT also causes an accumulation of guanidinoacetate (GAA), which is neurotoxic [7–11]. Recent data suggest that CTD has an estimated global prevalence of about 1 in 225,000 individuals worldwide, whereas GAMT may affect approximately 1 in 540,000 people [12, 13]. However, these numbers are likely an underestimate.

CTD and GAMT both cause cerebral creatine deficiency and as such, there are similarities between the two conditions in how they biologically affect the brain and also in their manifestations. Both CTD and GAMT typically present during infancy and are often characterized by intellectual and/or developmental disabilities, frequently accompanied by behavior problems, delayed or absent speech, seizures, motor impairments, and feeding and weight gain issues, consistent with the phenotype observed in other genetic neurodevelopmental conditions [7, 14–19]. For CTD, most literature is based on males, and heterogeneity is evident [17]. Heterozygous females with CTD have been reported to generally exhibit fewer symptoms [14], but as more females are being diagnosed, this gap in the literature can be addressed. Early diagnosis also contributes to patient heterogeneity. Evidence from GAMT sibling studies and early-treated infants has shown that starting treatment before symptom onset can lead to improved developmental outcomes, demonstrating that the condition’s severity can vary depending on the timing of diagnosis and intervention [20]. Diagnostic approaches for CTD and GAMT include biochemical analyses of urine and plasma, magnetic resonance spectroscopy (MRS), and genetic testing to confirm a cerebral creatine deficiency [14]. Early detection of these conditions is paramount, as timely intervention can significantly enhance treatment efficacy and patient outcomes.

Although in CTD, partial response to oral supplementation of creatine and creatine precursors (such as arginine and glycine), has been reported in single patients [5], there is still no treatment effectively tackling the trajectory of developmental intellectual disability and related caregiver burden [21, 22]. Pathophysiologically this relates to creatine’s inability to effectively cross the blood–brain barrier [23] and limitations in intracerebral creatine synthesis [24]. CTD patients currently utilize symptomatic therapies (e.g., antiepileptic medications, speech and occupational therapies) to improve functioning. For GAMT, oral creatine supplementation has shown efficacy in partially restoring brain creatine levels while dietary arginine restriction and L-ornithine supplementation may further reduce GAA levels [25–30]. The current management for GAMT requires lifelong treatment with a protein restricted diet and intake of high amounts of supplements [27, 31, 32]. In both CTD and GAMT, timely diagnosis and early intervention have been associated with enhanced quality of life and reduced cognitive impairment [2, 5, 26, 33]. Worldwide efforts are ongoing to understand the long-term impacts of CTD and GAMT [3, 34–37].

Implementing clinical trials in rare diseases such as CTD and GAMT is especially challenging due to factors that include a small population and substantial heterogeneity in phenotypes [38–42]. Identifying outcomes (e.g., clinical, behavioral, and/or laboratory-based) that assess treatment efficacy is critical to clinical trial success. Unfortunately, measuring different outcomes between trials makes it difficult to compare the efficacy of different therapeutics, especially when treatments target disease-modification of multi-systemic conditions affecting development [43, 44]. Historically, trial outcomes have been selected without input from patients and caregivers [45, 46]. Patients and caregivers are often the best equipped to identify the meaningful changes that would improve daily functioning and overall health as a result of a treatment. Recognizing this, the U.S. Food and Drug Administration (FDA) and other organizations now require patient and caregiver input as part of the clinical trial design process [47]. To achieve this, the FDA Patient-Focused Drug Development (PFDD) process released a four-part guidance, and this project is responsive to at least the first three: methods for systematic and meaningful inclusion of patient input [48], best practices for identifying disease and symptom elements that matter most to patients [49], and selecting clinical outcome assessments (COA) that measure outcomes that are important to patients [50].

A core outcome set (COS) can be used to address these obstacles to rare disease research and inform trial designs for treatments that are developed. A COS is a small set of outcomes that are established as important to be collected in every clinical trial of the same disease in order to systematically compare outcomes that are deemed important to patients and families [51, 52]. A COS promotes efficient use of resources and minimizes risk of bias by incorporating stakeholder perspectives, thereby ensuring that the evidence generated addresses outcomes that reflect meaningful treatment responses [53]. It also facilitates uniformity in the selection, measurement, and reporting of outcomes to simplify comparison across studies [53, 54]. A COS supports the generation of patient-centered evidence and its translation into policy and clinical practice, ensuring that researchers are prepared to evaluate therapies effectively when they become available [55, 56]. Having an established COS may be useful for clinical trial readiness for researchers examining potential clinical outcome assessments, as they can rely on the COS as a foundation given that we incorporated patient and caregiver perspectives from the beginning. To support the design of future clinical trials, we established the first COS for CTD and GAMT. 

## Methods

### Project design

We relied on the Core Outcome Measures in Effectiveness Trials (COMET) Initiative to guide us in developing our COS [53, 57, 58]. Our findings are reported in accordance with the COS Standard for Reporting statement (Supplemental 1) [59].

**Fig. 1:**
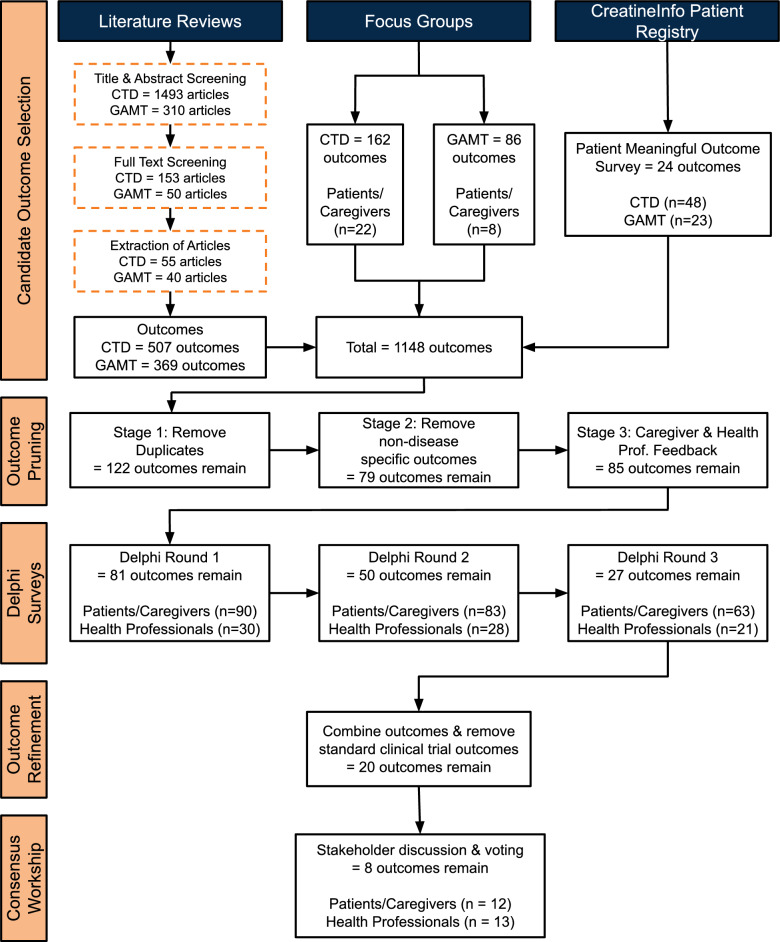
Outcome selection process for COS development for CTD and GAMT. During the candidate outcome selection phase, the total number of candidate outcomes gathered at each stage were identified. During the project's remaining phases, the total number of remaining outcomes at the end of each stage were identified. The total number of CTD and GAMT responses from the CreatineInfo data were identified, along with the total number of patients/caregivers and health professionals from the focus groups, Delphi surveys, and consensus workshop

To ensure that the final COS reflected the patient and caregiver perspective, active patient and caregiver engagement was a key design element throughout the COS development process. The project was conducted in three sequential phases: 1) Candidate Outcome Selection, 2) Delphi Surveys, 3) Consensus Workshop (Fig. 1).

### Patient and caregiver engagement

CTD and GAMT result in severe neurodevelopmental disorders, with affected individuals often nonverbal. Since caregivers must therefore serve as the patient’s voice, caregivers were critical in representing lived experiences. An active partnership between caregivers, clinicians, researchers, and scientists was utilized. This project was a component of a broader Patient-Centered Outcomes Research Institute^®^ (PCORI^®^)-funded patient engagement project “Parents Advancing REsearch NeTworkS” or PAReNts [60]. The PAReNts project aimed to empower caregivers and build their capacity to partner with researchers. Broadly, members of the PAReNts project received training and education about clinical trial research and COS development, shared their personal experiences, participated in focus groups, and actively engaged with other stakeholders to help develop the final COS for CTD and GAMT.

### Candidate outcome selection

The goal of the candidate outcome selection phase was to identify possible outcomes for CTD and GAMT from a variety of sources and diverse perspectives. The sources were as follows:

#### Rapid literature reviews

Two separate rapid literature reviews were conducted, one for CTD and one for GAMT, following the Cochrane rapid review methodology (Supplemental 2, Supplemental 3) [61]. The protocols for the CTD and GAMT reviews were published on PROSPERO [62] and Open Science Framework (OSF) [63], respectively. Embase (via OVID) and MEDLINE (via OVID) were searched for relevant articles. Article screening, carried out on Covidence [64], involved two reviewers (ZNM, JL) who independently screened the title, abstract, and full-text of each article. Articles not meeting the inclusion criteria (Supplemental 4) were excluded. Conflicts that could not be resolved between the reviewers were discussed with a third reviewer (SS-I). Data relating to outcomes, bibliographic information, and population details were extracted by one reviewer and verified by another.

#### CreatineInfo patient registry & natural history study

Additional outcomes were identified from the CreatineInfo Patient Registry and Natural History Study [65]. CreatineInfo is a Cerebral Creatine Deficiency Syndrome (CCDS) patient- and caregiver-reported registry and natural history study sponsored and managed by the Association for Creatine Deficiencies (ACD), hosted on the National Organization for Rare Disorders^®^ (NORD^®^) IAMRARE^®^ Registry platform. Outcomes were inspected from de-identified, aggregate data shared from CreatineInfo’s previously conducted Patient Meaningful Outcomes (PMO) survey which asked participants to identify and rate the outcomes that were most important to them [66].

#### Focus groups

The focus groups aimed to identify outcomes important to patients and caregivers that may be underrepresented in existing research and therefore missed by the rapid literature reviews. In-person and virtual semi-structured focus groups were conducted with caregivers of individuals with CTD and GAMT. In-person focus groups took place at ACD’s annual symposium while virtual focus groups were conducted separately. Participants were recruited through their affiliation with the “Creatine Deficiency Support Group'' and/or the “ACD Family Network”. One interpreter was present in one of the in-person focus groups.

Focus group participants were grouped according to proband diagnosis (CTD and GAMT) and age (0–8 years old and 9 + years old), when possible, for a total of five focus groups (three in-person and two virtual). Each focus group included three project team members: a facilitator who guided discussion and ensured equitable speaking time for each participant, an observer who served as quality control to the process and identified any bias that was introduced by the project team, and a notetaker. Participants discussed a series of broad questions designed to elicit their views on outcomes that would be important to them if improved with a treatment or therapy, based on their lived experiences (Supplemental 5).

The focus groups were audio-recorded and transcripts were generated using Otter.ai. The transcripts were cross-referenced with the audio recordings, and all identifying information was removed. Familiarization with the data was achieved through repeated, thorough readings of the transcripts, during which all patient- and caregiver-reported outcomes were extracted, capturing the essence of participants’ statements. Two project team members (ZNM, EKR) independently extracted outcomes from the focus group transcripts. To ensure consistency in the extraction process, both team members reviewed the analysis work of the other to confirm that outcomes were identified and extracted using a shared approach. The extracted outcomes were then refined by two team members (ZNM, SS-I) through an iterative process that involved removing duplicates and reaching team consensus on the grouping of similar outcomes. These unique outcomes were subsequently mapped to the relevant COMET Initiative’s outcome domains, consistent with the approach used in the literature review [67]. This process enabled comparison between outcomes identified through caregiver input and those reported in the published literature, while also ensuring the inclusion of additional outcomes uniquely identified in the focus groups.

#### Outcome pruning

The focus group outcomes were added to the list of candidate outcomes from the rapid literature reviews and CreatineInfo. The combined list underwent several stages of refinement by two project team members (ZNM, SS-I), who critically assessed the uniqueness of each outcome and applied a priori exclusion criteria. Outcomes were excluded if they were deemed irrelevant for inclusion in a COS, specifically if they were non-disease specific and/or extremely rare. This judgement was informed by SS-I’s extensive CCDS clinical expertise. Following this initial refinement, the remaining project team members and health professionals further refined the list by removing outcomes they considered unsuitable for inclusion in a COS for CTD and GAMT and suggesting outcomes they felt were missing. Clear definitions for the remaining outcomes were then developed by the project team in consultation with the PAReNts project cohort and health professionals.

### Delphi surveys

The goal of the Delphi survey phase was to systematically reduce the list of candidate outcomes by asking the CCDS community to rate and rank the importance of candidate outcomes [68]. We conducted three Delphi survey rounds to collect data on which outcomes are most important to participants. We used the findings to move toward consensus on the list of candidate outcomes for our final COS.

#### Delphi survey development

Three sequential rounds of Delphi surveys (“Round 1”, “Round 2”, and “Round 3”) were created to evaluate the importance of candidate outcomes. Data were collected and managed using REDCap (Research Electronic Data Capture) hosted at Vanderbilt University [69, 70]. In all Rounds, participants were asked, for each outcome, “How important do you think it is for research studies to measure [outcome]” on a scale of 1–9, with 1–3 being of “limited importance”, 4–6 being “important but not critical”, and 7–9 being “most important”. Open text boxes were available in Rounds 1 and 2 for additional feedback. Caregivers rated each outcome based on their lived experience with the disorder (either CTD or GAMT), while health professionals could specify which candidate outcomes applied to each of the disorders, based on their clinical expertise. In Rounds 2 and 3, each outcome was accompanied by graphical distributions of ratings from the previous round by “Patients and Caregivers” and “Health Professionals”, and returning participants were provided with their own previous rating. This process facilitated consensus on the outcomes, which is a key element of the Delphi methodology [71]. In Round 3, participants also ranked their top 10 most important outcomes. Each survey included two catch trials that participants had to answer correctly for their entry to be included in the analysis. The surveys used lay language which was reviewed and improved through PAReNts review for clarity. This facilitated comprehension among survey participants. Surveys were available in English, French, and Spanish, with translations completed by CryaCom International Inc. translation services and reviewed by native speakers.

#### Delphi eligibility and recruitment

Adult patients (18 years and older) who were able to express themselves and parents/caregivers of patients with CTD or GAMT were eligible to participate (“Patients and Caregivers”). Professionals with experience working with individuals with CTD and/or GAMT were also eligible to participate (“Health Professionals”). This included clinicians, researchers, laboratory scientists, health policy advisors, and additional professionals and therapists (e.g., speech therapists, psychologists, teachers). Round 1 was open to all eligible participants. Round 2 was open to Round 1 participants and new eligible participants. Round 3 was open exclusively to Round 1 and Round 2 participants.

Participants were recruited through direct email, newsletters, meetings, webinars, and social media. Recruitment materials were available in English, French, and Spanish. All recruitment materials for patients and caregivers were reviewed in advance by the PAReNts project caregivers. Active recruitment varied by survey round but lasted between 4 and 8 weeks per round. Participants received weekly email reminders to finish their surveys. Surveys were accessed via a publicly available link (Round 1) or by personalized survey links (Rounds 2 and 3).

#### Delphi survey analysis

**Fig. 2:**
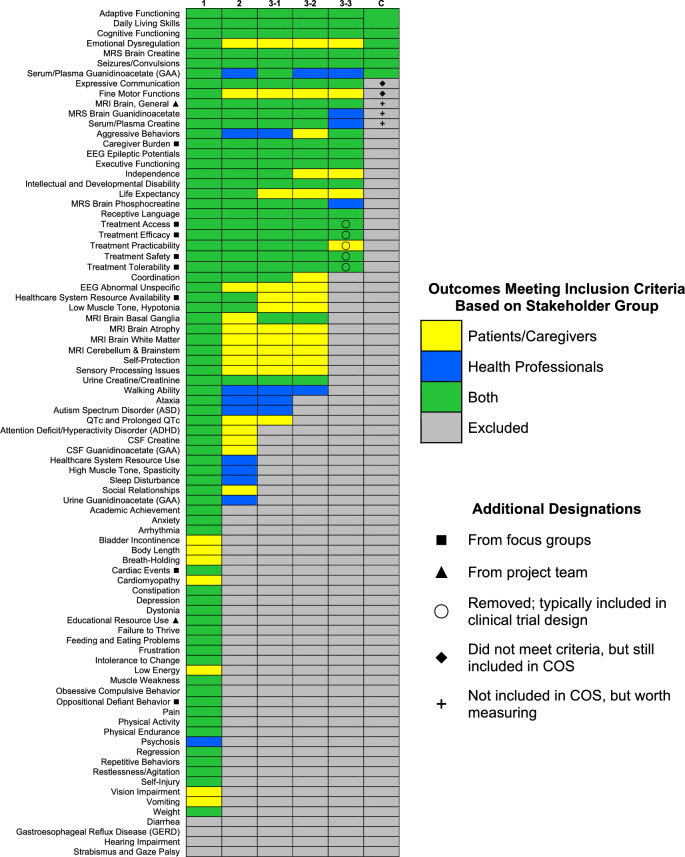
Outcomes remaining after each Delphi survey round and the consensus workshop. Column headers reflect the Delphi round and each set of inclusion criteria. The inclusion criteria for each round were as follows: Delphi 1, rated ≥ 3 by ≥ 70% of any stakeholder group; Delphi 2, rated ≥ 7 by ≥ 70% of any stakeholder group; Delphi 3–1, rated ≥ 7 by ≥ 70% of any stakeholder group; Delphi 3–2, mean rating ≥ 7 for any stakeholder group; Delphi 3–3, ranked in top 10 by ≥ 15% of any stakeholder group; Consensus (C), ≥ 50% of workshop participants voted outcome as “1-Definitely In”. Stakeholders meeting the inclusion criteria for each outcome are identified by color: patients/caregivers (yellow), health professionals (blue), both patients/caregivers and health professionals (green), outcome did not meet the criteria and was excluded (gray). Some outcomes were combined and/or changed throughout the process: “Developmental Delay” and “Intellectual Disability” were combined into “Intellectual and Developmental Disability”, “Adaptive Functioning” and “Daily Living Skills” were combined into “Adaptive Functioning”, and “Expressive Language” was changed to “Expressive Communication”. Outcomes marked with ◼ came exclusively from the focus groups. Outcomes marked with ▲ were introduced by the project team during the pruning phase. Outcomes marked with ◯ were removed by the project team because they are typically already included in clinical trial designs. Outcomes marked with ◆ did not meet the consensus workshop inclusion criteria but were later included after unanimous agreement among participants. Outcomes marked with + are not included in the COS, but are worth measuring alongside the COS

At the completion of each survey round, the data were analyzed to determine which candidate outcomes were most important among stakeholders. Those outcomes that garnered the most support by reaching stakeholder consensus advanced to the next round. Stakeholder consensus was defined by the outcome inclusion criteria in each round, with the criteria becoming more rigorous in subsequent rounds. Inclusion criteria specifics are found in Fig. 2. Criteria were applied first to “Patients and Caregivers” and “Health Professionals”; if the criterion was met for either of these groups, the outcome advanced. If an outcome did not meet the inclusion criterion for at least one of these two stakeholder groups, the subgroups “CTD Patients and Caregivers” and “GAMT Patients and Caregivers” were analyzed separately. An outcome had to meet the criterion for one of these subgroups to advance. In Round 3, inclusion criteria were applied in a stepwise manner (Fig. 2; i.e., 3–1, 3–2, 3–3) in which an outcome had to meet all three criteria to advance. Upon completion of Round 3, the remaining list of candidate outcomes were further refined by combining similar outcomes and removing non-disease specific outcomes.

### Consensus workshop

The goal of the consensus workshop was to prioritize the remaining candidate outcomes and reach consensus on a final list of 8–10 core outcomes that would become the COS for CTD and GAMT. The workshop was held in person at the University of Utah, Salt Lake City, Utah, USA; one member of the project team attended virtually. A group of 25 participants with a diverse range of perspectives attended the workshop to facilitate a holistic discussion of outcomes. Workshop participants consisted of CTD caregivers, GAMT caregivers, and health professionals/researchers including those who would use the COS in a clinical and research setting. The majority of workshop participants had completed at least one round of the Delphi survey and most of the caregivers were PAReNts cohort members. Two separate pre-workshop meetings were held for caregivers and health professionals to prepare participants for successful engagement in the workshop.

The workshop began with an overview of the COS development project and a review of Round 3 results. Utilizing an adapted Nominal Group Technique [72], each participant was then given one minute to provide their perspective on their three most important outcomes. Guided by a neutral moderator (BKP), an open discussion unfolded among workshop participants. After thorough discussion of outcomes, each participant voted on the candidate outcomes via a Google Form. Caregivers voted once based on their lived experience with the disorder, while health professionals voted once for CTD and once for GAMT, based on their expertise. There were three response choices for each candidate outcome:

1. 1-Definitely In: outcome must be in the COS
2. 2-Maybe: outcome is a strong contender for the COS
3. 3-Definitely Out: outcome can be left out of the COS in favor of higher priority outcomes

Participants were asked to select a maximum of 5 outcomes as “1-Definitely In”. Once all participants had voted, the data were organized by disorder, analyzed using the consensus inclusion criteria, and shared with participants. Workshop participants discussed the voting results and eventually reached consensus on a final set of core outcomes.

## Results

### Candidate outcome selection

#### Rapid literature reviews

A total of 1803 articles were screened across both CTD (*n* = 1493) and GAMT (*n* = 310). After full-text screening, a total of 203 articles were retained for CTD (*n* = 153) and GAMT (*n* = 50). From these, 95 articles met the inclusion criteria (Supplemental 4) and were used for outcome extraction for both CTD (*n* = 55) and GAMT (*n* = 40). This process yielded a total of 876 outcomes identified through the CTD (*n* = 507) and GAMT (*n* = 369) rapid literature reviews (Fig. 1).

#### CreatineInfo patient registry & natural history study

Data from the CreatineInfo Patient Registry Patient Meaningful Outcomes (PMO) survey yielded 24 outcomes reported as important to patients and caregivers (*n* = 71, Fig. 1). All 24 outcomes were reported by both CTD (*n* = 48) and GAMT (*n* = 23) participants.

#### Focus groups

Thirty patients and caregivers participated across five focus groups, with three CTD groups (n = 22) and two GAMT groups (n = 8). A total of 248 outcomes were generated from the CTD (n = 162) and GAMT (n = 86) focus groups (Fig. 1). The complete list of unique candidate outcomes identified during the focus groups is presented in Supplemental 6.

#### Outcome pruning

At the completion of the candidate outcome selection (i.e., literature review, CreatineInfo Patient Registry, and focus groups) 1148 outcomes were identified. Following the outcome pruning stage (i.e., removal of duplicates and non-disease-specific outcomes), 122 outcomes remained. After incorporating feedback from caregivers and health professionals, this list was further refined to 85 candidates and their definitions.

Most of the 85 candidate outcomes were identified from multiple sources. Interestingly, 23 of the 85 outcomes were only found in the literature reviews, while eight were unique to the focus groups and not identified from any of the other sources. There were no unique outcomes identified only from the CreatineInfo Patient Registry. Of the 85 outcomes, two emerged from discussions with the project team during the outcome pruning stage (Fig. 1).

### Delphi surveys

**Fig. 3:**
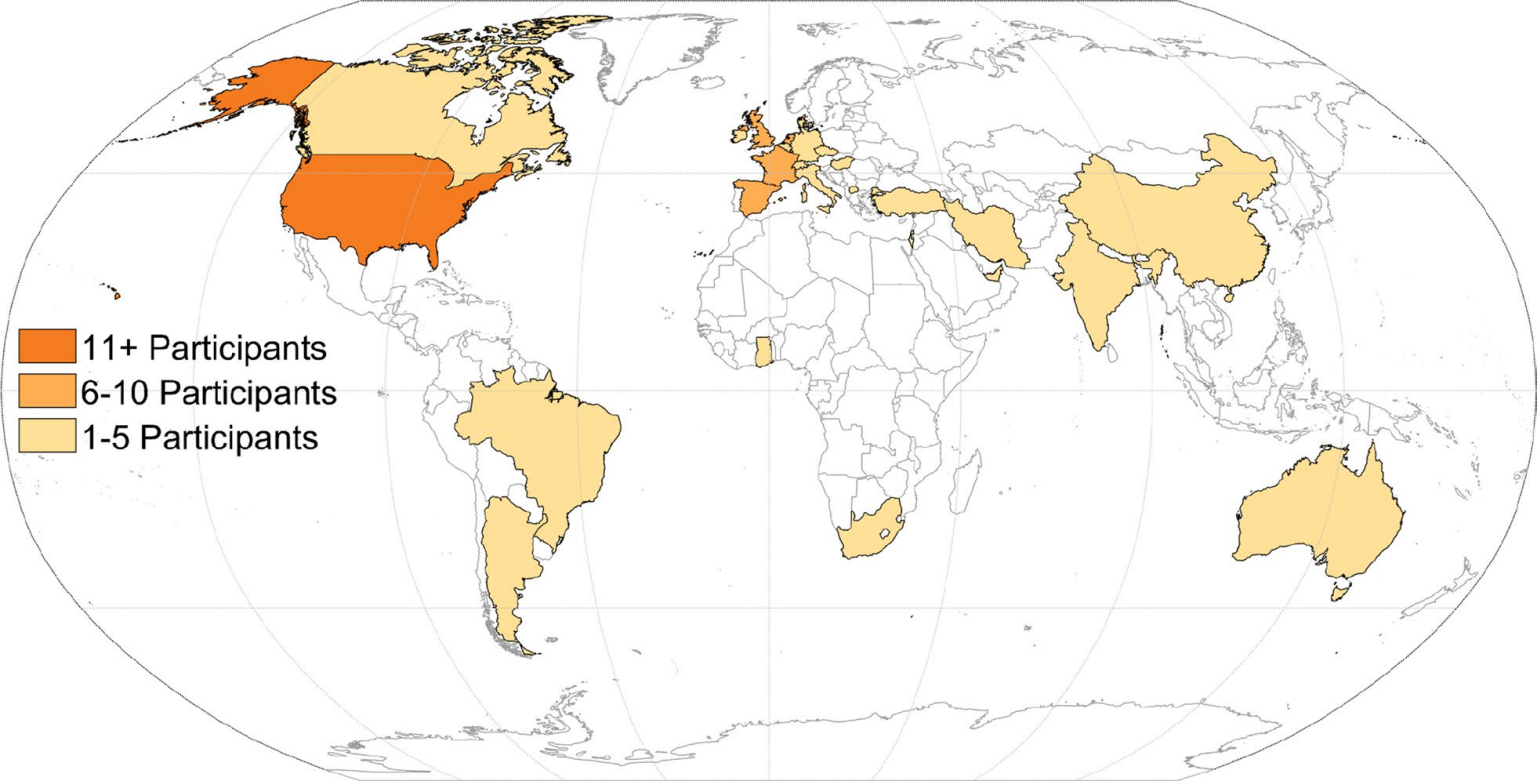
Distribution of locations for the participants (n = 150) who completed the Delphi surveys. Represented countries were categorized into three groups: 1–5 participants (light orange, n = 22), 6–10 participants (orange, n = 4), and 11 or more participants (dark orange, n = 1)

Collectively, 150 individuals participated in the Delphi surveys from 27 countries (Fig. 3) and six continents (Table 1). In Round 1, 120 participants rated 85 candidate outcomes. In Round 2, 81 remaining outcomes were rated by 111 participants, 32 of whom were new participants in Round 2 (i.e., retention rate from Round 1 was 71.2%). In Round 3, 84 participants rated the remaining 50 outcomes; 66 of the 84 participants (78.6%) had participated in Round 1, and 75 of the 84 participants (89.3%) had participated in Round 2. In each Delphi round, participants who did not answer at least 25% of the outcome survey questions or failed to correctly answer the catch trials were excluded from analysis. After Round 3, we further refined the candidate outcomes; similar outcomes were combined and five additional outcomes were removed because they are already typically included in clinical trial designs (Fig. 2). No new outcomes were identified from comments shared by participants in open text boxes during Rounds 1 and 2. Detailed aggregate results from the Delphi survey are presented in Supplemental 7.

**Table 1:** Demographics of focus groups, Delphi surveys, and consensus workshop participants

	Focus Groups n (%)	Delphi Round 1 n (%)	Delphi Round 2 n (%)	Delphi Round 3 n (%)	Consensus Workshop n (%)
**Patients/caregivers of children with CTD or GAMT**	n = 30	n = 90	n = 83	n = 63	n = 12
*Diagnosis*					
CTD	22 (73)	72 (80)	64 (77)	51 (81)	7 (58)
GAMT	8 (27)	18 (20)	19 (23)	12 (19)	5 (42)
*Gender*					
Woman	–	58 (64)	53 (64)	36 (57)	–
Man	–	30 (33)	24 (29)	23 (37)	–
Unspecified	–	2 (2)	6 (7)	4 (6)	–
*Age treatment began (GAMT only)*					
Before 2 years of age	–	7 (39)	7 (37)	5 (42)	–
Age 2 years or later	–	11 (61)	9 (47)	6 (50)	–
*Continent*					
Africa	–	3 (3)	3 (4)	3 (5)	–
Asia	–	5 (6)	3 (4)	4 (6)	–
Australia	–	2 (2)	2 (2)	1 (2)	–
Europe	–	26 (29)	18 (22)	14 (22)	–
North America	–	45 (50)	46 (55)	33 (52)	–
South America	–	4 (4)	3 (4)	2 (3)	–
Unspecified	–	5 (6)	8 (10)	6 (10)	–
*Race/Ethnicity* ^*a*^					
Asian	–	3 (3)	4 (5)	4 (6)	–
Black	–	2 (2)	3 (4)	3 (5)	–
Hispanic, Latino, or Spanish origin	–	7 (8)	5 (6)	3 (5)	–
Middle Eastern or North African	–	2 (2)	1 (1)	2 (3)	–
White	–	72 (80)	64 (77)	47 (75)	–
Some other race, ethnicity, or origin ^b^	–	0 (0)	1 (1)	1 (2)	–
Prefer not to answer	–	3 (3)	1 (1)	1 (2)	–
Unspecified	–	2 (2)	6 (7)	4 (6)	–
**Health professionals**		n = 30	n = 28	n = 21	n = 13
*Gender*					
Woman	–	21 (70)	19 (68)	13 (62)	–
Man	–	7 (23)	5 (18)	6 (29)	–
Prefer not to say	–	1 (3)	1 (4)	1 (5)	–
Unspecified	–	1 (3)	3 (11)	1 (5)	–
*Continent*					
Asia	–	2 (7)	2 (7)	2 (10)	–
Europe	–	6 (20)	7 (25)	6 (29)	–
North America	–	21 (70)	17 (61)	13 (62)	–
Unspecified	–	1 (3)	2 (7)	0 (0)	–
*Race/Ethnicity *^*a*^					
Asian	–	5 (17)	3 (11)	3 (14)	–
Hispanic, Latino, or Spanish origin	–	0 (0)	2 (7)	0 (0)	–
Middle Eastern or North African	–	1 (3)	1 (4)	1 (5)	–
White	–	21 (70)	19 (68)	17 (81)	–
Some other race, ethnicity, or origin ^b^	–	1 (3)	1 (4)	1 (5)	–
Prefer not to answer	–	3 (10)	2 (7)	1 (5)	–
Unspecified	–	0 (0)	1 (4)	0 (0)	–
*Health Professional Role *^*a*^					
Biochemical geneticist, metabolic specialist, geneticist	–	12 (40)	10 (36)	9 (43)	–
Developmental neurologist, neurologist	–	3 (10)	5 (18)	3 (14)	–
Pediatrician	–	2 (7)	1 (4)	1 (5)	–
Physiotherapist, physical therapist, speech therapist, occupational therapist	–	3 (10)	2 (7)	2 (10)	–
Psychologist	–	1 (3)	2 (7)	1 (5)	–
Teacher	–	2 (7)	0 (0)	0 (0)	–
Other ^c^	–	4 (13)	2 (7)	1 (5)	–
Unspecified	–	1 (3)	1 (4)	0 (0)	–
*Employment setting *^*a*^					
Academic institution	–	19 (63)	14 (50)	13 (62)	–
Healthcare setting (e.g., hospital, private clinic)	–	13 (43)	12 (43)	10 (48)	–
Hospital-based research institute	–	3 (10)	4 (14)	4 (19)	–
Other ^d^	–	6 (20)	1 (4)	0 (0)	–
Unspecified	–	1 (3)	1 (4)	1 (5)	–

^a ^Participants had the option to select more than one answer

^b ^Additional responses included: “Cuban”, “Turkish”

^c ^Additional responses included: “Behavior analyst”, “ABA therapist”, “Pediatric Epileptologist”

^d^ Additional responses included: “ABA Organization”, “Home and School”

### Consensus workshop

Ahead of the consensus workshop, the 20 candidate outcomes were categorized into four core areas to highlight the comprehensive nature of our remaining candidate outcomes: “Life Impact”, “Growth and Development”, “Physiological/Clinical”, and “Death” (Table 2) [67, 73, 74].

**Table 2:** Candidate outcomes discussed during the consensus workshop

Core area & definition	Outcomes discussed during consensus workshop (*n* = 20)	Included in final COS?
Life Impact A general concept of well-being that refers to a combination of various life aspects (e.g., health, emotional, social), as well as caregivers’ perspectives about their involvement and observations	Aggressive behaviors	
	Caregiver burden	
	Emotional dysregulation	Yes
	Seizure/convulsions	Yes
Growth and Development The process of change across a combination of physical, emotional, cognitive, social, and language areas over the course of childhood	Adaptive functioning	Yes
	Cognitive functioning	Yes
	Executive functioning	
	Expressive communication^a^	Yes
	Fine motor functions	Yes
	Independence	
	Intellectual & developmental disability	
	Receptive language	
Physiological/Clinical Observable and measurable abnormal changes and symptoms in the body as a result of CTD/GAMT	EEG epileptic potentials	
	MRS brain creatine	Yes
	MRI brain general	
	MRS brain guanidinoacetate	
	MRS brain phosphocreatine	
	Serum/plasma creatine	
	Serum/plasma guanidinoacetate	Yes
Lifespan/Death The length/end of a person’s life	Life expectancy	

^a^Outcome was originally called “Expressive Language” but was changed to “Expressive Communication” during the consensus workshop

Consensus workshop participants reviewed, discussed, and voted on the 20 candidate outcomes (Supplemental 8). Six outcomes were voted as “1-Definitely In” by more than 50% of workshop participants and were included in the COS: “Adaptive Functioning”, ”Cognitive Functioning”, “Emotional Dysregulation”, “MRS Brain Creatine”, “Seizure/Convulsions”, and “Serum/Plasma Guanidinoacetate” (for GAMT specifically). Two outcomes, “Expressive Communication” and “Fine Motor Functions”, did not meet the inclusion criteria but were included based on unanimous agreement during the group discussions. Thus, a total of eight outcomes (seven for CTD and GAMT, and one additional outcome for GAMT) were defined for the CTD and GAMT COS, with their definitions shown in Table 3. Three outcomes that were excluded from the COS were identified by workshop participants as worth tracking as they are often measured in parallel with the required COS: “MRI Brain General”, “MRS Brain Guanidinoacetate'', and “Serum/Plasma Creatine” (Fig. 2).

**Table 3:** The final core outcome set (COS) for CTD and GAMT

Core outcome	Definition
Adaptive functioning	An individual’s level of independence in functioning compared to similarly-aged peers, in areas including communication and practical tasks, such as daily living skills involving toileting, eating, dressing, and hygiene
Cognitive functioning	Specific mental abilities, including the ability to learn, think, remember, problem solve, as well as decision-making, and attention
Emotional dysregulation	Having a difficult time appropriately managing and controlling one’s feelings and emotional responses
Expressive communication	Ability to express one’s needs and wants through communication
Fine Motor Functions	Motor skills that involve the smaller muscles (e.g., wrists, hands, fingers) and allow for more precise movement
MRS Brain Creatine ^a^	Amount of creatine in the brain as determined by magnetic resonance spectroscopy (MRS), a technique that shows the levels of chemical components in the brain
Seizure/Convulsions	Sudden and uncontrollable movements and/or loss of consciousness and/or loss of body control; episodes can be self-limiting and last for a few seconds, or they can persist, or come in clusters
Serum/Plasma Guanidinoacetate^ b^	Amount of guanidinoacetate in the serum or plasma

Consensus workshop participants provided comments on two of the eight outcomes:

^a^ “MRS Brain Creatine” is worth noting in GAMT clinical trials. However, it is anticipated that this biomarker may remain stable if the patient received oral creatine supplementation prior to the trial

^b^ “Serum/Plasma Guanidinoacetate” should be measured in GAMT clinical trials, but is not an appropriate outcome in CTD trials

During the consensus workshop discussion, participants removed the outcomes “Intellectual & Developmental Disability”, “Receptive Language”, “Independence”, and “Executive Functioning”. While “Intellectual & Developmental Disability” is a prominent diagnosis in the population, its components, “Cognitive Functioning” and “Adaptive Functioning”, were included in the COS and can be measured with metrics more sensitive to change. “Independence” is reflected in “Adaptive Functioning” while “Executive Functioning” is an aspect of “Cognitive Functioning”. “Expressive Language” was renamed “Expressive Communication” to include non-verbal forms of communication.

## Discussion

Using a structured consensus process, we successfully identified a set of eight core outcomes that should be used in clinical trials and research studies for CTD and GAMT based on caregiver and health professional input. Following the recommended COMET framework, we identified candidate outcomes by performing literature reviews, focus groups, and Delphi surveys, and finalized the COS by holding an in-person consensus workshop [53]. The final COS includes outcomes from three of the relevant recommended core areas, meeting the current recommendation that a COS be holistic to better represent the patient [73, 74].

Seven of the eight outcomes are applicable to both CTD and GAMT. However, one of the biomarker outcomes, “Serum/Plasma Guanidinoacetate” is a unique outcome exclusive to GAMT because elevated GAA levels are a hallmark of GAMT, making GAA a responsive biomarker for GAMT treatment. Similarly, measurement of creatine with magnetic resonance spectroscopy (MRS), “MRS Brain Creatine”, is recommended for CTD clinical trials as cerebral creatine is markedly low in CTD and may serve as a responsive biomarker for treatment. However, this outcome is also recommended for monitoring in the case of GAMT since it may be normalized in patients receiving oral creatine supplementation prior to a trial with a new therapy. It is also worth noting that for both of these biomarker outcomes, they would likely not serve as surrogate endpoints in trials, but should be studied for their potential correlations with the six other outcomes in the COS that relate to clinical outcomes. As of this time, there is some indication that “Serum/Plasma Guanidinoacetate” and “MRS Brain Creatine” are related to neurodevelopmental outcomes but more data needs to be collected to further understand the link between these biomarkers and outcomes related to everyday functioning [29].

Workshop participants agreed that three outcomes eliminated from the core outcome set will often be measured in parallel with the required COS and are worth tracking. In the case that plasma is collected for “Serum/Plasma Guanidinoacetate”, tracking “Serum/Plasma Creatine” is encouraged in trials that aim to replenish GAMT activity and thus endogenous synthesis of creatine. Similarly, when measuring “MRS Brain Creatine”, also measuring “MRS Brain Guanidinoacetate'' may provide valuable monitoring insights in GAMT trials. When magnetic resonance imaging (MRI) is performed in parallel with MRS, any abnormality in “MRI Brain General” in GAMT and CTD clinical trials is recommended to be noted. This is supported by the fact that certain abnormalities (e.g. abnormal signal intensity in basal ganglia) are reversible in patients with GAMT upon treatment with creatine and/or guanidinoacetate lowering therapies [28, 75].

### Caregiver involvement in developing a COS for two rare conditions

CTD and GAMT present with similar phenotypes and share the treatment goal of restoring creatine to the brain [14]. Additionally, patients, caregivers, researchers, and clinicians actively partner together to advance research on these two conditions. Given their ultra-rare nature, both conditions benefit from mutual support within this collaborative community. We hypothesized that very few critical differences in prioritized outcomes would be identified between these two conditions, thereby driving our effort to develop a COS for CTD and GAMT simultaneously. Since seven of the core outcomes were applicable to both conditions, based on the Delphi process as well as the consensus workgroup, evidence is provided for this hypothesis, although it is possible that combining parts of the consensus workshop may have influenced the consensus in decisions. However, it is also worth noting that in addition to these two conditions, a recent review of many genetic conditions associated with neurodevelopmental disorders found outcomes in these areas were amongst the most commonly used [76].

Though we utilized multi-stakeholder collaboration to develop a COS, we took deliberate measures to ensure that caregiver and patient voices remained distinct. Our caregiver focus groups resulted in eight candidate outcomes that were exclusively identified by caregivers, with one outcome (“Caregiver Burden”) receiving extensive discussion during the consensus workshop (see Fig. 2). By conducting caregiver focus groups and analyzing Delphi responses separately for each diagnosis and stakeholder group, we created a COS that is informed by the experiences of CTD and GAMT patients and families. For example, the impacts of “Seizures/Convulsions”, a frequent characteristic of both CTD and GAMT [14], were highlighted during the consensus workshop when one caregiver conveyed the effects on their family:

*“Ninety-nine percent of my burden is my daughter’s seizures and her moods that come out of nowhere because she’s going to have a seizure. And that they ruin my family’s life."*

Similarly, many caregivers expressed concern about their child(ren)’s “Emotional Dysregulation”, an outcome that encompasses an array of emotional responses such as aggression and irritability. One caregiver expressed during the focus group:

*“Last night, I had an iPad thrown at my face. He gets mad really quickly. He can't seem to control his outbursts. [He’s] put his foot through a wall before.”*

Impaired “Expressive Communication” was another key concern of many caregivers. During the focus group, one parent shared their frustration when their child is unable to verbally communicate with others:

*"I can see that he gets disappointed when he cannot get his message across to others. And it's frustrating for me because I'm trying to understand him, but I can't.”*

These real-world examples demonstrate how caregivers’ experiences and their related outcomes are captured in the COS. Patient and caregiver participation in our project was integral to developing a COS that is not only disease-specific but also patient-centered.

While consensus on outcomes was eventually reached, differences in outcome prioritization between caregivers and health professionals were identified during the Delphi survey and consensus workshop phases (Fig. 2). For example, “Emotional Dysregulation” and “Fine Motor Functions” both met the inclusion criteria among caregivers, but not health professionals, in Delphi Rounds 2 and 3. While “Emotional Dysregulation” met the consensus workshop inclusion criteria, “Fine Motor Functions” initially did not. After further discussion, workshop participants unanimously agreed to include both “Emotional Dysregulation” and “Fine Motor Functions” in the final COS. Conversely, “Serum/Plasma Guanidinoacetate (GAA)” met the inclusion criteria among health professionals, but not caregivers, in Delphi Rounds 2 and 3. During the consensus workshop discussions, participants reached a unanimous agreement to include it in the final COS. Additionally, “Caregiver Burden” was an outcome that received thoughtful debate among consensus workshop participants. Caregivers argued that improvements in patient-focused outcomes will naturally lead to reduced “Caregiver Burden”, making inclusion of this outcome unnecessary. Health professionals agreed to defer to caregivers and this outcome was not included in the COS.

Caregivers utilized their research engagement training to effectively collaborate with health professionals and advocate for their families during the consensus workshop, directly influencing the final COS. For instance, caregivers voiced their concern that “Expressive Language” and its original definition was too limited and exclusionary, failing to capture meaningful improvements in non-verbal forms of communication (e.g., sign language, communication devices). Participants collectively agreed to change this outcome to “Expressive Communication” and modify the definition to include non-verbal forms of communication. Throughout the COS development process, caregivers’ lived experiences complemented the health professionals’ clinical expertise, resulting in a COS for CTD and GAMT that will serve as a starting point but will be re-evaluated and studied further for prioritization and clinical meaningfulness.

### COS importance in CTD and GAMT clinical trials

Given the rarity of CTD and GAMT, this COS will become increasingly impactful as new treatments are developed, as the COS represents an opportunity to ensure that therapies are systematically evaluated based on meaningful outcomes. Additionally, using the COS reduces waste, standardizes comparison across studies, and promotes collaboration and data sharing. We expect our COS to increase the efficiency of research methods and clinical trial designs, and ultimately expedite the study of treatments for CTD and GAMT. Moreover, regulatory agencies, such as the U.S. FDA, can be reassured that patient perspectives are included in the proposed trial design when the COS is included. By including patient and caregiver perspectives in establishing a COS for CTD and GAMT, the investigator burden of creating patient-centered trial design is greatly reduced.

### Recommendations for COS development and future directions

This collaboration marks the first COS developed for CTD and GAMT, and is one of only a few efforts to create a COS for inborn errors of metabolism. Our approach shares many similarities with other COS development projects for inborn errors of metabolism, including mucopolysaccharidoses (MPS) [77, 78], phenylketonuria (PKU) and medium-chain acyl-coenzyme A dehydrogenase (MCAD) deficiency [79], as they were also guided by the COMET framework. Our project is unique compared to other COS development projects, as it incorporated all of the following design elements: (1) caregiver and health professional participation, (2) focus groups, (3) literature reviews, (4) patient registry data, (5) Delphi surveys, and (6) a consensus workshop. Other unique features instrumental to our consensus process included progressively more rigorous Delphi inclusion criteria, approximately equal numbers of caregiver and health professionals in the consensus workshop, and our workshop voting process.

We hope that other disease populations, especially other rare diseases, will benefit from the strengths of our COS development project and our recommendations on potential areas for improvement as they develop their own COS. One of the unique components of this project was the active engagement of caregivers in the entire process, as part of our larger PAReNts project. Caregivers received training and education in COS and clinical trial development, learned how to become better advocates by sharing their personal stories, and contributed to the focus groups, Delphi surveys, and consensus workshops. Health professionals commented on how knowledgeable caregivers were during the workshop discussions:

*“Their ability to synthesize and comment on the scientific issues was amazing.”*

Based on our experience, we propose the use of adaptable inclusion criteria for retainment of proposed outcomes between Delphi survey rounds. Many patients and caregivers find it challenging to deprioritize outcomes [53], which may result in limited variability in the scoring of outcomes using less rigorous a priori criteria. After thoughtful consideration, we implemented more rigorous post hoc criteria in Delphi Rounds 2 and 3 to filter the total number of outcomes. Reducing outcomes prior to survey distribution decreases the burden on participants and the attrition rate between rounds [53, 80].

We reflect that the pre-workshop training and the in-person format of our consensus workshop, with approximately equal numbers of caregivers and health professionals in attendance, helped us reach consensus on the COS more efficiently. Additionally, the alternating placement of stakeholders throughout the consensus workshop room facilitated open conversation with all participants and encouraged them to make new connections.

Future COS development projects should prepare for potential challenges. In the case of stakeholder participation, it is important to target recruitment of stakeholders who are from traditionally underrepresented groups to facilitate their participation and minimize bias. For example, GAMT is rarer than CTD; therefore, we employed various recruitment strategies to ensure a more representative participant pool between the two conditions, while still not reaching equal representation. We recommend inclusion of health policy advisors, as we believe this would have provided us with valuable insights on clinical trials during this project. It is important to consider in advance how to accommodate the communication needs of participants. Our materials only included English, French, and Spanish translations; additional languages would have broadened participation. It is important to consider translating recruitment documents, project materials, and the Delphi surveys into multiple languages, and providing interpretation services or closed captioning during focus groups and the consensus workshop.

While the outcomes established in the COS serve as a foundation, we acknowledge that the measurement of additional outcomes may be beneficial depending on the type of therapeutic intervention, characteristics of the target patient population, and other variables. For instance, phenotypes of males and females with CTD may vary significantly and warrant the inclusion of additional outcomes to capture these differences. An N-of-1 trial may benefit from additional customized outcomes as well. All of these considerations speak to the need to continue to utilize the existing CreatineInfo registry data, analyze data from natural history data collections, and acquire additional data to better understand longitudinal trajectories in CTD and GAMT. This information about the conditions will help formulate appropriate understanding of what to expect for endpoint changes or the need to consider different outcomes.

Understanding how to measure the COS is an important next step. Outcomes may be measured using different tools. Not all tools may be effective in a particular population, especially among neurodevelopmentally impacted patient populations like CTD and GAMT. Development of patient-centered considerations for selecting outcome measurement tools appropriate to this patient community is a logical next step and will serve as a valuable companion to our COS [53].

## Conclusion

In order to design appropriate and relevant studies and clinical trials for rare diseases, it is important to have a group of evidence-based and patient-centered core outcomes that are consistently measured to enable comparison across studies and facilitate clinical trials. Here, we share the first COS for CTD and GAMT. Throughout the development of this COS, patient and caregiver perspectives were considered through their engagement in focus groups, Delphi surveys, and the consensus workshop. We especially benefited from the caregivers who partnered with us to co-design the recruitment materials, outcome definitions, Delphi surveys, and attended the consensus workshop. This COS is a step towards designing more appropriate clinical trials and accelerating the development of effective interventions for CTD and GAMT.

## Supplementary Information

Additional file 1.

Additional file 2.

## Data Availability

Raw data are not publicly available due to privacy and/or ethical restrictions. Aggregate data that supports the findings of this study are available in the supplementary materials of this article. Additional data may be made available on request from the corresponding author. Data ownership is maintained by ACD.

## Abbreviations

ACD: Association for Creatine Deficiencies
CCDS: Cerebral creatine deficiency syndromes
COA: Clinical outcome assessments
COMET: Core Outcome Measures in Effectiveness Trials
COS: Core outcome set
CTD: Creatine transporter deficiency
FDA: Food and Drug Administration
GAA: Guanidinoacetate
GAMT: Guanidinoacetate methyltransferase
MRI: Magnetic resonance imaging
MRS: Magnetic resonance spectroscopy
NORD^®^: National Organization for Rare Disorders^®^
OMT: Outcome measurement tool
OSF: Open Science Framework
PAReNts: Parents Advancing REsearch NeTworkS
PCORI^®^: Patient-Centered Outcomes Research Institute^®^
PFDD: Patient-focused drug development
PMO: Patient meaningful outcomes
REDCap: Research Electronic Data Capture
