# Supplementary material for: Establishing a core outcome set for creatine transporter deficiency and guanidinoacetate methyltransferase deficiency
Source: Orphanet J Rare Dis. 2025 Aug 7;20:408. doi: 10.1186/s13023-025-03900-3 (PMC12333098; doi:10.1186/s13023-025-03900-3)
Supplement: Supplementary file 1 — Additional file 1. [file 13023_2025_3900_MOESM1_ESM.docx]

# **SUPPLEMENTARY MATERIAL**

## Supplemental 1. Core Outcome Set-STandards for Reporting: The COS-STAR Statement Checklist

| **Section/Topic** | **Item No.** | **Checklist Item** | **Reported On Page Number** |
| --- | --- | --- | --- |
| Title/Abstract | | | |
| Title | 1a | Identify in the title that the paper reports the development of a COS | 1 |
| Abstract | 1b | Provide a structured summary | 1 |
| Introduction | | | |
| Background and Objectives | 2a | Describe the background and explain the rationale for developing the COS. | 2-3 |
|  | 2b | Describe the specific objectives with reference to developing a COS. | 2-3 |
| Scope | 3a | Describe the health condition(s) and population(s) covered by the COS. | 2-3 |
|  | 3b | Describe the intervention(s) covered by the COS. | 2-3 |
|  | 3c | Describe the setting(s) in which the COS is to be applied. | 2-3 |
| Methods | | | |
| Protocol/Registry Entry | 4 | Indicate where the COS development protocol can be accessed, if available, and/or the study registration details. | 3 |
| Participants | 5 | Describe the rationale for stakeholder groups involved in the COS development process, eligibility criteria for participants from each group, and a description of how the individuals involved were identified. | 3, 5-6 |
| Information Sources | 6a | Describe the information sources used to identify an initial list of outcomes. | 3-5, Figure 1 |
|  | 6b | Describe how outcomes were dropped/combined, with reasons (if applicable). | 4-6, Figures 1-2 |
| Consensus Process | 7 | Describe how the consensus process was undertaken. | 5-8, Figure 1 |
| Outcome Scoring | 8 | Describe how outcomes were scored and how scores were summarised. | 5-8, Figure 2 |
| Consensus Definition | 9a | Describe the consensus definition. | 6 |
|  | 9b | Describe the procedure for determining how outcomes were included or excluded from consideration during the consensus process. | 5-8, Figures 1-2 |
| Ethics and Consent | 10 | Provide a statement regarding the ethics and consent issues for the study. | 15 |
| Results | | | |
| Protocol Deviations | 11 | Describe any changes from the protocol (if applicable), with reasons, and describe what impact these changes have on the results. | 14 |
| Participants | 12 | Present data on the number and relevant characteristics of the people involved at all stages of COS development. | 6, 8, 10-11, Table 1, Figure 3 |
| Outcomes | 13a | List all outcomes considered at the start of the consensus process. – table 2 | 11, Table 2, Supplemental 7 |
|  | 13b | Describe any new outcomes introduced and any outcomes dropped, with reasons, during the consensus process. | 8-9, 12, Table 2 |
| COS | 14 | List the outcomes in the final COS. | 8, 12, Table 3 |
| Discussion | | | |
| Limitations | 15 | Discuss any limitations in the COS development process. | 14 |
| Conclusions | 16 | Provide an interpretation of the final COS in the context of other evidence, and implications for future research. | 14 |
| Other Information | | | |
| Funding | 17 | Describe sources of funding/role of funders. | 15 |
| Conflicts of Interest | 18 | Describe any conflicts of interest within the study team and how these were managed. | 15 |

Note: This table was sourced from the paper published by Kirkham et al. (2016) which provides guidance for reporting of all COS studies [59].

##

## Supplemental 2. Rapid literature review outcome extraction sheet

##
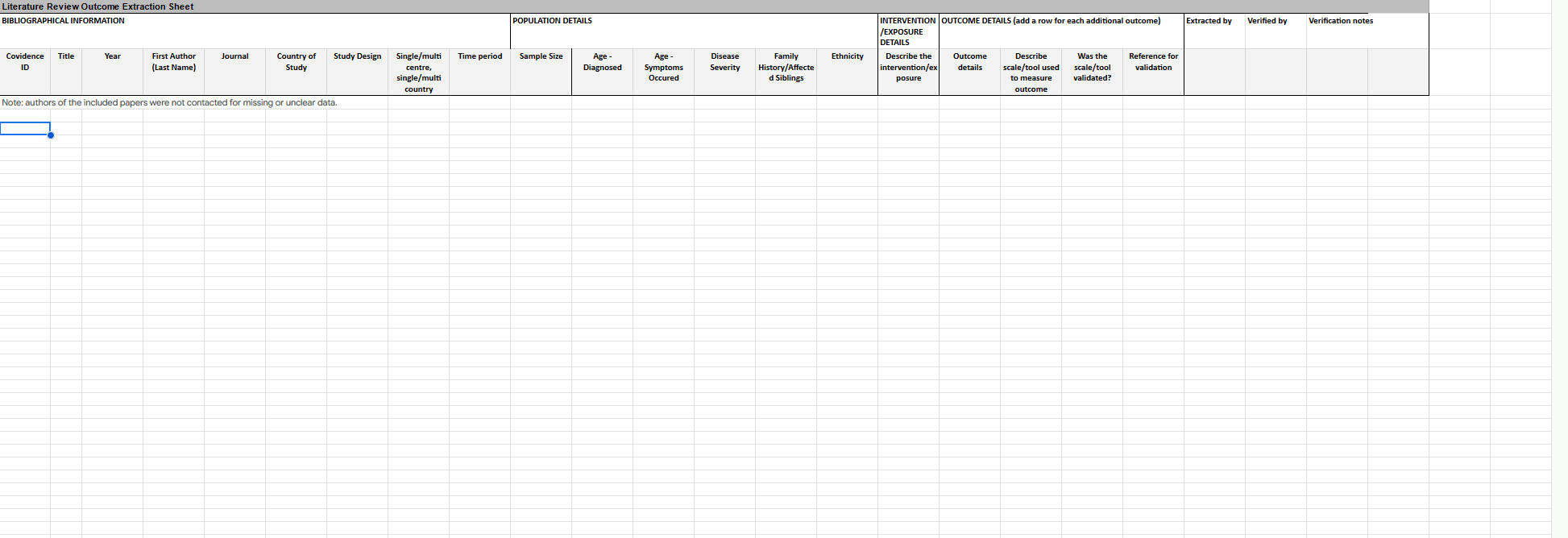


Note: This supplemental file includes the extraction sheet used to record outcomes identified through our CTD and GAMT literature reviews

##

##

## Supplemental 3. Rapid literature review search strategies

| **CTD Search Strategy** | |
| --- | --- |
| Set | Search Statement |
| 1 | *Mental Retardation, X-Linked/ or *Creatine/ |
| 2 | (CRTD or creatine transporter* deficienc* or creatine transporter* activit*).mp. |
| 3 | SLC6A8*.mp. |
| 4 | 1 or 2 or 3 |
| 5 | patient reported outcome measures/ |
| 6 | Outcome Assessment, Health Care/ |
| 7 | Treatment Outcome/ |
| 8 | ((patient* or core) adj3 (outcome* or measure*)).tw,kf. |
| 9 | (outcome* adj3 (assessment* or treatment* or therapy*)).tw,kf. |
| 10 | (prom or proms).tw,kf. |
| 11 | or/5-10 |
| 12 | Intellectual Disability/ or Developmental Disabilities/ or Developmental Delay/ |
| 13 | (intellect* disabilit* or learn* disabilit* or development* disabilit* or development* delay* or development* regress*).tw,kf. |
| 14 | Epilepsy/ or Seizures/ or Seizures, Febrile/ |
| 15 | (epilep* or seizure*).tw,kf. |
| 16 | (speech adj3 (delay* or impediment*)).tw,kf. |
| 17 | Movement Disorders/ or Muscle Hypotonia/ or Muscle Weakness/ or "Activities of Daily Living"/ |
| 18 | (movement disorder* or muscle hypotonia* or muscle weakness* or activit* of daily living*).tw,kf. |
| 19 | (dystonia* or spasticit* or parkinsonism* or rigidit* or ataxia* or chorea* or asteatos*).tw,kf. |
| 20 | dementia*.tw,kf. |
| 21 | Autism/ or ADHD/ or ASD/ |
| 22 | (autis* spectrum disorder* or autism or attention* deficit* hyperactivity* disorder*).tw,kf. |
| 23 | Problem Behavior/ |
| 24 | (problem adj3 (behaviour* or behavior*)).tw,kf. |
| 25 | Mental Disorders/ or Anxiety/ or Depression/ or OCD/ or Schizophrenia/ |
| 26 | (mental disorder* or mental illness* or anxiety or depression or obsessive compulsive disorder* or OCD or schizophrenia).tw,kf. |
| 27 | Insomnia/ or Apnea/ or Parasomnia/ or Nocturnal Enuresis/ |
| 28 | (insomnia* or apnea* or parasomnia* or nocturnal enuresis*).tw,kf. |
| 29 | Feeding Behavior/ |
| 30 | (feeding adj3 (behaviour* or behavior*)).tw,kf. |
| 31 | Pain Measurement/ or Pain Management/ or Pain/ |
| 32 | (pain* or pain tolerance or hyperacusis or sensory processing disorder or food avers* or hypersensitiv* or hyperalgesia).mp. [mp=title, abstract, heading word, drug trade name, original title, device manufacturer, drug manufacturer, device trade name, keyword heading word, floating subheading word, candidate term word] |
| 33 | Vision/ or Hearing/ or Sensory/ or Somatosensory Disorders/ |
| 34 | (vision or hearing or sensory).tw,kf. |
| 35 | Osteoblasts/ or Osteoporosis/ or Bone Diseases/ |
| 36 | (bone density or osteopenia or osteoporosis or osteoblast* or bone disease*).tw,kf. |
| 37 | Gastrointestinal Diseases/ |
| 38 | (gastrointestinal disease* or gastrointestinal symptom* or constipation or acid reflux or GERD or gastroesophageal reflux disease).tw,kf. |
| 39 | (weight or height).tw,kf. |
| 40 | "Quality of Life"/ |
| 41 | (quality of life or life quality).tw,kf. |
| 42 | exp Communication Disorders/ |
| 43 | communicat*.tw,kf. or expressive language delay*.mp. or speech delay*.mp. or delay in speech.mp. or nonverbal communicat*.mp. or nonverbal.mp. or social communicat*.mp. or emotional communicat*.mp. or sign language.mp. or assisted communicat*.mp. or alternative communicat*.mp. or augumentative communicat*.mp. [mp=title, abstract, heading word, drug trade name, original title, device manufacturer, drug manufacturer, device trade name, keyword heading word, floating subheading word, candidate term word] |
| 44 | exp Sleep Wake Disorders/ |
| 45 | (sleep wake disorder* or sleep disorder* or sleep abnormalit* or insomnia* or parasomnia* or nocturnal enuresis or sleep disordered breathing or sleep disordered grinding or sleep apnea or restless sleep* or sleep restlessness or sleep restriction or hypersomnia).tw,kf. |
| 46 | or/12-45 |
| 47 | 11 or 46 |
| 48 | Creatine/ or phosphocreatine/ or exp Creatine Kinase/ or Biomarkers/ |
| 49 | (Creatine or phosphocreatine or creatine phosphate or creatine kinase or creatine phosphokinase or Brain creatine or urine creatine or guanidinoacetate or CK or CPK).tw,kf. |
| 50 | 48 or 49 |
| 51 | 4 and 47 |
| 52 | 4 and 47 and 50 |
| **GAMT Search Strategy** | |
| Set | Search Statement |
| 1 | Guanidinoacetate N-Methyltransferase/ |
| 2 | (guanidinoacetate methyltransferase deficien* or GAMT deficien* or cerebral creatine deficiency).mp. |
| 3 | (creatine adj3 (deficien* or disorder*)).mp. |
| 4 | 1 or 2 or 3 |
| 5 | intellectual disability/ or developmental disability/ or developmental delay/ or developmental disabilities/ |
| 6 | ((intellectual* or neurologic* or mental* or cerebral* or neurodevelopment* or neuro-development* or development* or learn* or cognitive or cognition) adj3 (impair* or dysfunction* or delay* or disabilit* or disorder* or regress* or deficit*)).mp. |
| 7 | (global developmental delay* or GDD).mp. |
| 8 | epilepsy/ or seizures/ or seizure, febrile/ |
| 9 | (epileps* or epileptic encephalopath* or seizure*).mp. |
| 10 | Dementia/ |
| 11 | dementia*.mp. |
| 12 | Mental Disorders/ or Anxiety/ or Depression/ or OCD/ or Schizophrenia/ |
| 13 | (mental disorder* or mental illness* or anxiety or depression or obsessive compulsive disorder* or OCD or schizophrenia or depressed or anxious or compulsive behavior* or compulsive behaviour*).mp. |
| 14 | Autism/ or Autism Spectrum Disorder/ or ASD/ or ADHD/ |
| 15 | (autism* or attention deficit* hyperactiv* disorder* or ADHD or ASD).mp. |
| 16 | Problem Behavior/ |
| 17 | ((aggression or aggressive or problem*) adj3 (behaviour* or behavior*)).mp. |
| 18 | ((abnormal or self-mutilat* or self mutilat* or selfmutilat* or auto-mutilat* or auto mutilat* or automutilat* or self-injur* or self injur*) adj3 (behavior* or behaviour*)).mp. |
| 19 | Communication Disorders/ |
| 20 | (communicat* or expressive language delay* or speech delay* or speech impediment* or delay in speech or nonverbal or non-verbal or social communicat* or emotional communicat* or sign language or ASL or assist* communicat* or alternat* communicat* or augumentat* communicat*).mp. |
| 21 | ((Absent or slurred) adj3 (speech or language)).mp. |
| 22 | ((speech or language) adj3 (impediment* or delay* or impairment*)).mp. |
| 23 | (no language or no speech).mp. |
| 24 | Feeding Behavior/ |
| 25 | ((feed* or eat*) adj3 (behaviour* or behavior*)).mp. |
| 26 | Movement Disorders/ or Muscle Hypotonia/ or Muscle Weakness/ or "Activities of Daily Living"/ |
| 27 | ((motor or movement*) adj3 (impair* or dysfunction* or delay* or disabilit* or disorder* or regress* or deficit*)).mp. |
| 28 | (stiff posture* or abnormal posture*).mp. |
| 29 | (dysarthri* or hyperreflexia* or hyper-reflexia* or spasticit* or spastic or ataxia* or ataxic or dystoni* or hypotoni* or hypo-toni* or Parkinson* or Pes Cavus or choreoathetosis or choreo-athetosis).mp. |
| 30 | (myopath* or neuropath*).mp. |
| 31 | (delay* adj3 (sit* or stand* or walk*)).mp. |
| 32 | ((muscle* or muscular) adj3 (reduc* or decrease* or atroph* or weakness or hypotoni* or hypo-toni*or dystoni* or dystroph*)).mp. |
| 33 | (activit* of daily living* or ADL*).mp. |
| 34 | arthritis/ |
| 35 | (arthrit* or osteoarthrit* or osteo-arthrit*).mp. |
| 36 | Osteoblasts/ or Osteoporosis/ or Bone Diseases/ |
| 37 | (bone fracture* or bone deformit* or osteoporosis or osteopenia* or bone densit* or osteoblast* or bone disease*).mp. |
| 38 | Sleep Wake Disorders/ or Insomnia/ or Apnea/ or Parasomnia/ or Nocturnal Enuresis/ |
| 39 | (sleep wake disorder* or sleep-wake disorder* or sleep disorder* or sleep abnormalit* or insomnia* or parasomnia* or nocturnal enuresis or sleep disordered breathing or sleep disordered grinding or sleep apnea or restless sleep* or sleep restlessness or sleep restriction* or hypersomnia* or apnea).mp. |
| 40 | (extrapyramidal syndrome or extrapyramidal dysfunction or involuntary movement* or drool* or salivat*).mp. |
| 41 | chorea*.mp. |
| 42 | Pain Measurement/ or Pain Management/ or Pain/ |
| 43 | (pain* or pain toleran* or hyperacusis or sensory processing disorder* or food avers* or hypersensitiv* or hyperalgesi*).mp. |
| 44 | Vision/ or Hearing/ or Sensory/ or Somatosensory Disorders/ |
| 45 | (vision or hearing or sensory or somatosensor* or somato-sensor*).mp. |
| 46 | Gastrointestinal Diseases/ |
| 47 | (gastrointestinal disease* or gastro-intestinal disease* or gastrointestinal symptom* or gastro-intestinal symptom* or constipat* or diarrhea* or acid reflux* or GERD or gastroesophageal reflux diseas* or gastro-esophageal reflux disease* or growth retard* or failure to thrive or short stature).mp. |
| 48 | body height/ or body weight/ |
| 49 | (body weight or body height or head circumference*).mp. |
| 50 | "Quality of Life"/ |
| 51 | (quality of life or quality-of-life or life quality or life-quality or QOL).mp. |
| 52 | or/5-51 |
| 53 | Biomarkers/ |
| 54 | (Biomarker* or biochemical marker* or test* or assess* or GAA or guanidinoacetate or creatinine or Glycine or MRI or MRS or magnetic resonance imaging or magnetic resonance spectroscopy or elevated GAA* or elevated level* of GAA or serum creatine or GAA level* or biochemical assay*).mp. |
| 55 | Creatine/ |
| 56 | (creatine adj3 (supplement* or excret* or deplet*)).mp. |
| 57 | patient reported outcome measures/ or Outcome Assessment, Health Care/ or Treatment Outcome/ |
| 58 | (patient reported outcome measure* or patient outcome measure* or PROM* or POM* or outcome assessment*).mp. |
| 59 | or/53-58 |
| 60 | 4 and 52 |
| 61 | 59 and 60 |

Note: The final search strategies for the CTD and GAMT rapid literature reviews. Embase (via OVID) and Medline (via OVID) were used to search for relevant papers.

## Supplemental 4. Inclusion and exclusion criteria for the rapid literature reviews

| **Inclusion Criteria** |
| --- |
| - Subjects in the testing group of studies must be diagnosed with CTD or GAMT via genetic testing or biochemical confirmatory testing (e.g., brain creatine levels, creatine uptake studies, urine, plasma and CSF creatine or GAA levels, etc.). |
| - Biomarkers, surrogate outcomes, medically important and patient/caregiver meaningful outcomes will be used as primary outcome measures. |
| - Assessment tool/instrument (MRI, MRS, etc.) is described/outlined. |
| **Exclusion Criteria** |
| - Full text not available online. |
| - Experimental/animal studies. |
| - Publication not available in English. |
| - Published protocols. |
| - Subjects diagnosed with other neurodevelopmental disorders in addition to CTD/GAMT (e.g., additional chromosomal abnormality). |

Note: Inclusion and exclusion criteria for CTD and GAMT rapid literature reviews. Covidence was used for title/abstract and full text screening.

## Supplemental 5. Focus group questions

1. What do you think are desirable outcomes that should be measured as part of a treatment study for children with CTD/GAMT? These could be important to you, your child, or both.
2. From the perspective of your child, what are the biggest challenges that they suffer from most?
3. From your perspective as a caregiver, what are the biggest challenges you face because of your child’s creatine deficiency?
4. What do you want to see happen as an outcome of treatment?
5. What are your concerns or worries about a drug trial?
6. Are there outcomes that you feel are so ideal, you expect they are not achievable through a drug trial?

## Supplemental 6. Candidate outcomes identified during the focus groups

| **COMET Core Area** | **Outcome Domain** | **Definition** | **Outcome** | **Example Quotes** |
| --- | --- | --- | --- | --- |
| Physiological or Clinical | Blood and lymphatic system outcomes | Measures of physiological function, signs and symptoms, and laboratory measures relating to blood and lymphatic system outcomes | - Improved blood circulation (Resolve red - column splotches, especially on legs and bright red ears) | “[My child] has...poor circulation and tolerance for temperature extremes...he won't eat food that's more than warm, he won't eat ice cream...his bath has to be very lukewarm and things like that” (CTD) |
|  | Cardiac outcomes | Measures of physiological function, signs and symptoms, and laboratory measures relating to cardiac outcomes | - Reduced cardio events - Evaluate the frequency and different types of cardio events - Decrease in cardio issues - Normal heart rate | “Decreased low heart rate, or too high, he goes back and forth. And right now we're in this period of his heart rate’s a little bit too high. And that also seems to increase the seizures. But they're not finding anything. So I don't know what's causing that” (CTD) |
|  | Congenital, familial, and genetic outcomes | Measures of physiological function, signs and symptoms, and laboratory measures relating to congenital, familial, and genetic outcomes | - Early detection | “I honestly question how much the intellectual development, the cognitive ability, can be improved in a drug trial. I would love it if it could - in my heart and soul [I] would. And I know that's a big one. But I have serious doubts that the damage isn't already done.” (CTD) |
|  | Ear and labyrinth outcomes | Measures of physiological function, signs and symptoms, and laboratory measures relating to ear and labyrinth outcomes | - Improved auditory processing | “He wants everything super loud” (CTD) |
|  | Musculoskeletal and connective tissue | Measures of physiological function, signs and symptoms, and laboratory measures relating to musculoskeletal and connective tissue | - Orthotics - Degree of scoliosis - Equinus contractures - Other miscellaneous orthopedic conditions - Being able to walk - Walk longer distances - Improved overall strength and muscle strength - Gain muscle weight - Neck control | ““He…has low physical stamina. He's not able to go as much as he likes walking with us, but he just gets tired sometime[s].” (GAMT)  “His strength, his muscle strength. He wants to be stronger.” (GAMT) |
|  | Nervous system outcomes | Measures of physiological function, signs and symptoms, and laboratory measures relating to nervous system outcomes | - Reduction in seizures - Quality, frequency, and quantity of seizures   - Fewer/no absent seizures   - Decreased amount of epileptic activity, normal EEG   - Normal brain functions eg. epilepsy background on EEG as opposed to absent seizures   - Improvement in the side effects caused by seizure meds   - Resolve “emotional seizures”   - Resolve tonic clonic and laughing seizures   - Resolve life-threatening seizures   - Seizures without enuresis/bedwetting - Decreased breath-holding spells - Improved sensory processing - Improved motor abilities (gross motor) - Be able to sign (use sign language) - Reduced clumsiness - Improved coordination - Mobility - Level of abnormal gait - Toe walking - Improved muscle tone - Improved fine motor function - Normalized pain tolerance (Eg. not high) being able to detect pain, increased sensitivity to pain - Improved temperature tolerance (hands and feet turn red) - Less sensitive to temperature changes, less sensitive when taking showers, increased sensitivity to auditory stimuli - Resolve seizures and dystonia - Improved hypertonia and hypotonia - Improved MRI or MRS - Decreased tactile diversions - Stay dry overnight - Improved sensory stimulation - Lowering or normalizing the guanidinoacetate | "And it's so painful, and it affects everybody. It affects all of us…I believe once the seizures [are] no more, he will be able to focus, he will be in good health." (CTD)  "My son developed seizure[s]…[at] a couple months old. And they call it…a breath-holding spell…so anytime the temperature change[d] or he [laid] down on his back. And now, he's progressed to tonic-clonic...laughing seizures.” (CTD)  “Clumsy - unable to walk correctly. Fall[s] down; you don't have that coordination“ (CTD)  “Improved muscle tone” (GAMT)  “Some good measurable things I can think of definitely lowering, normalizing the [GAA]“ (GAMT) |
|  | Gastrointestinal outcomes | Measures of physiological function, signs and symptoms, and laboratory measures relating to gastrointestinal outcomes | - Improved GI issues - Resolve constipation - Constipation without throwing up - Frequency of cyclic vomiting - Frequency of vomiting - Decreased vomiting - Resolve aspirations - GI motility - Delayed stomach emptying - Level of acid reflux | "He eats all right, but I think initially he used to vomit a lot. And he has aspirations, and all that it took a while, I think after a year before it stopped. Now the vomit has reduced drastically. But still there are feeding difficulties with the weight gain." (CTD)  "He eats all right, but I think initially he used to vomit a lot. And he has aspirations, and all that it took a while, I think after a year before it stopped. Now the vomit has reduced drastically. But still there are feeding difficulties with the weight gain." (CTD) |
|  | Metabolism and nutrition outcomes | Measures of physiological function, signs and symptoms, and laboratory measures relating to metabolism and nutrition outcomes | - Keep weight on - Weight gain - Feed well - Normal diet - Liquid calorie rich dietary supplementation - Less restricted diet - Be able to eat protein (protein restriction is hard for the child) - Eliminate (drastic) weight loss - Not be significantly underweight - No more G tube - Improved energy - Resolve feeding difficulties | “And when he was 19, he started losing weight, like nobody's business, like lost like 50 pounds, and was then starting to have behaviors” (GAMT) |
|  | Renal and urinary outcomes | Measures of physiological function, signs and symptoms, and laboratory measures relating to renal and urinary outcomes | - Not having to use a diaper | “He goes to the toilet quite automatically. We don't have to tell him and it works all day and [some] days [it] doesn't work at all. So if we go out of the house, we always use diapers and even with diapers…[it] seems like he doesn't care.” (GAMT) |
|  | Psychiatric outcomes | Outcomes include all those relating to mental health conditions and associated behaviors (e.g. addictions and behavioral problems). | - Impulse control and decreased impulsivity - Improved emotional regulation and processing - Decreased irritability - Social and emotional anxiety - Reduced anxiety and stress levels - Reduced self-harm - Reduced refusal (going limp) - Decrease in hitting, throwing - Decreased frustration - Sleep deprivation for caregiver and patient, eg. codependent sleeping, improved quality of sleep - Reduced hyperactivity - Decreased aggressiveness, outbursts of anger and refusal - Be able to control outbursts - Decreased oppositional defiant behaviors. - Improved social and emotional maturity - Consistent pleasant behaviors, good behavior - Decreased stereotypies, decreased hand movements or flapping or gesturing - Decreased need for routine - Decreased overstimulation and being able to calm his nervous system, (eg. with a sibling, in a care home, etc.) - Less agitation | “See a decrease in certain behaviors, the hitting, the throwing the, the impulsivity, the anxiety, those pieces, I think would also be desirable outcomes.” (CTD)  “Handle emotions like without engaging in self injurious behaviors, be able to be around others appropriately.”  (CTD)  “One thing that [my child] still has is the stereotypies which are the hand movements or flapping or she does a lot of gesturing with her fingers. And so…improvement in the stereotypies.” (GAMT) |
| Life impact | Physical functioning | Physical functioning: impact of disease/condition on physical activities of daily living (for example, ability to walk, independence, self-care, performance status, disability index, motor skills, sexual dysfunction. health behavior and management) | - Increased endurance, not getting tired quickly - Being able to drive - Improved functionality - Improved penmanship, proper grip - Hold a pen, draw - Improved physical stamina - Improved physical activity | “I think he knows he lacks some confidence. And all he really cares about [is] sports. So he is very aware of his performance and his ability to perform physically…he knows he's behind.“ (GAMT) |
|  | Cognitive functioning | Impact of disease/condition on cognitive function (e.g. memory  lapse, lack of concentration, attention); outcomes relating to knowledge, attitudes and  beliefs (e.g. learning and applying knowledge, spiritual beliefs, health beliefs/knowledge) | - Improved executive function and executive processing - Short-term working memory - Planning, execution - Improved cognitive function   - measured in IQ - Improved communication (including verbal)   - Communicate needs to people other than family and speech therapists, be able to explain his experiences in different environments (important in terms of safety and security)   - Interact appropriately with family and friends   - Improved ability to respond to others, improved responsiveness, reciprocate in conversation   - Interactive and more effective communication (give and take discussion)   - Have a normal conversation   - Don’t just communicate through questions   - Better communication with sibling   - Improved speech - Improved speech delivery and understanding - Improved functional speech and clarity of speech   - Improved expressive and receptive language - More precise or appropriate language, bigger vocabulary   - Speak any words   - Be able to speak a sentence with certain number of words - Resolve intellectual disability - Improved safety behavior and safety skills and vigilance - Sense danger - Increased ability to express pain (eg. constipation or other GI issues) - Improved behavior - Increased level of independence, be able to take care of himself - Be able to explore environment - Increased level of self-care, self-help   - Understand one's own needs and wants   - Improved desire for self-care and health care - Improved awareness of self-hygiene - Improved self-awareness - Improved self-play, self-directed activities - Be able to function as an adult - Improved daily living skills/tasks - Exhibiting a skill vs employing it   - Demonstrating the difference between capability and motivation   - Being able to demonstrate cognitive abilities - Educational success - Educational challenges - Better understand concepts in school - Improved focus and potential for learning, for behaviors not to impede learning - Ability to attend, focus on a task - Improved attention span/decreased attention deficit, be able to sit and perform tasks - Improved motor planning (including fine motor planning) - Decreased length of time and fewer repetitions required for learning new things. - Increased retention - Improved understanding and processing speed - Be able to count (To 10, or 100) - Improved reading comprehension - Improved overall comprehension - Comprehension of situations, situational awareness, display depth of understanding - Increased confidence and self-motivation - Improved flexibility and adaptability, don’t “freak out” if things don’t go as he/she had planned them. - Being able to plan for the future - Improved self-advocacy - Toilet trained and be able to wipe, cognitively understand the process - Be able to bathe self - Verbalize why he is stressed - Be able to act quickly - Take in information, express it, and act on it. - Decreased tendency to be easily distracted - Be able to stay in places where there’s a lot of people around - Resolve/prevent regression | “That confidence, that receptive, that ability to go back to processing, being able to take in the information and be able to express it and act on it and right, educationally and emotionally and it's the whole package…right?” (GAMT)  “When we're out in public places, even if it's with people that he sees on a regular basis are people that he's seen before, you know, other family members. If people he doesn't know come up to him and [try] to interact with him, he's very reserved...Other times, there's no boundaries. If I take him to a park or some[where] more fun, he just wants to take off and run. But he's not sensing any dangers that could arise. So that's, you know, an obvious issue to me. So he picks and chooses, you know, who he interacts with.” (CTD)  “I can put him under the shower, but he wouldn't wash himself. It's more a lack of understanding of washing, getting clean itself.” (CTD)  “If I put him in the care of somebody else...he's incredibly vulnerable. So if he were able to advocate for himself, somehow a drug allowed him more awareness for advocacy” (GAMT) |
|  | Social functioning | Impact of disease/condition on social functioning (e.g. ability to socialise, behavior within society, communication, companionship, psychosocial development, aggression, recidivism, participation) | - Improved social awareness - Build meaningful relationships - Appropriate social relations - Initiate social engagement - Be able to maintain and improve relationship with siblings - Appropriate behavior in the presence of a large group of people - Better understanding of social cues - Able to form friendships - Improved interaction with peers - Being able to have romantic relationships - Improved relationship | “He wants to interact with folks. He wants to form relationships. But he's just not able to have a normal give and take discussion. He asked questions of everyone. He knows the answers to them...That's his best effort to communicate, is to ask a question that he knows the answer to. So I know that bothers him.” (GAMT) |
|  | Role functioning | Impact of disease/condition on role (e.g. ability to care for children, work status) | - Have a job | “...able to function on his own and have, you know, friends and family…or [a] job even.” (CTD) |
|  | Global quality of health | Includes only implicit composite outcomes measuring global quality of life | - Improved quality of life - Improved overall health - Live a healthy long life - Have a normal functioning life - Enjoy life - Live like ordinary children | “Making sure they are healthy and will live a long life” (CTD)  “I really do desire…that my child can live like…other children like ordinary children” (GAMT) |
|  | Delivery of care | Includes outcomes relating to the delivery of care. | - Have a medical professional who is willing to prescribe treatment - Less time out of school to receive medical care - Easier access to treatment (worldwide) - Improved availability of ordering medications - More readily available treatment - Improved supplement taste/better tasting medicine - Not throw up when taking the medicine - Better drug delivery system. - Easier better medicine - Decreased irritability as a side effect of medication - Being able to swallow capsules - Simple once a day, palatable, liquid - More streamlined, less invasive treatment - Reduced dosage and frequency of treatment - Decreased reliance on supplements - Maintain treatment independently and improved compliance - Discrete treatment - Decreased overall struggle with supplements | “And no doctor is even, you know, willing to prescribe [treatment]. And I don't even know how to go about it.” (CTD)  “Taking her out of school so that she can go get a blood test because…the lab only takes blood during school hours and things like that…it's all that time spent out of school” (GAMT)  “The difficulty of access to the treatment” (GAMT)  “There's [a] shortage of ornithine or creatine, [they are] hard to get.” (GAMT)  “He hates his supplements. It's the taste, and specifically is the sodium benzoate…that's one of our biggest challenges is finding some type of delivery system...for all three of those, but specifically for ornithine. Because it's the one that [is] the hardest, the least palatable." (GAMT) |
| Resource use | Hospital | Outcomes relating to inpatient or day case hospital care (e.g. duration of hospital stay, admission to ICU) | - Reduce overall hospitalizations - Less hospitalization due to getting sick from not eating the right things | “Would overall hospitalizations be something we could measure? Yeah, that could get [at] quality of life a little bit, just just the general number of hospitalizations.” (CTD)  “Often these kids don't eat the right things and they get sick more often [so] they get hospitalized.” (CTD) |
|  | Societal/carer burden | Outcomes relating to financial or time implications on carer or society as a whole (e.g. need for home help, entry to institutional care, effect on family income) | - Caregivers/family not being isolated because of their child’s unpredictability in behavior - Have people over to eat - Not getting mad at caregivers - Not needing constant care - Not attaching to a particular caregiver. Let others come in and try to provide the care that they need. - Caregivers having more free time - Caregivers being able to give attention to each child in the family more equally - Adequate support and resources - Have long term care resources available when caregivers are not around any more - Being able to afford additional support and supplements - Caregiver being able to advocate for child in school - Reduced time weighing out of supplements and measuring of supplements, gathering supplies - Being able to understand what the child wants from caregivers - Less reliance on caregivers | "He attaches to one caregiver. And if anybody else comes into the picture…he doesn't want them getting in between him and his caregiver." (GAMT)  “My biggest challenges are worrying about my child functioning as an adult, the impact of the additional care time and energy that we need to support him, the impact on the schedule, the impact on our other children, and how he gets the most attention.” (GAMT)  "We're not always going to be here. Realistically, I know that he's probably never going to be able to live independently. But whether that's with one of his siblings, or in a group home, you know, I would want him to be able to control himself or, you know, to control his emotions and his behaviors in order to be able to function in that environment" (CTD) |
| Outcomes identified in focus groups that fall outside the established COMET framework | Need for further intervention | The need for additional medical, therapeutic, or pharmacologic support following the treatment or intervention* | - Fewer medical appointments and blood tests or investigations - Less reliance on various therapies (Eg. language and physical abilities) - Decrease reliance on seizure meds and other supplements (specifically ornithine) - Possibly no medication/treatment (eg. gene therapy) | “If there was a drug treatment that you wouldn't need to be taking the ornithine…[it] would be a more ideal outcome” (GAMT) |
|  | Outcomes that are too ideal | Outcomes that caregivers expect are too ambitious to be achievable through a drug trial* | - Normal/total improvement of intellectual development and cognitive ability - “Normal" abilities and intelligence - Living an independent life - Having a normal life - Full cognitive function - Becoming seizure-free. - Read and speak | “[My child having] kids, family, drive a car…friends for holidays. For me, it's just something that may not be achievable"  (CTD) |
|  | Concerns with a drug trial | Caregiver worries with any and all aspects of a drug trial* | - Availability of the drug trial to females - Availability of the drug in general - Adverse reaction to the drug or developing unknown or rare side effects - Preventing the participant from caring for their loved ones - Increase in seizure or epileptic activity or the return of seizures - Trial won't work - Not getting the drug trial | "I worried number one, that the trial won't work and that it would do additional unforeseen damage that we haven't anticipated because we had to correct brain damage, I'm worried that those corrections would be reversed. And then there might be new side effects that we aren't currently dealing with come as a result." (GAMT) |

Note: Focus group outcomes were categorized by the COMET core areas and outcome domains [67]. Some outcomes identified during the focus group did not fit into the published COMET framework. Therefore, new outcome domains and definitions (marked with *) were developed to capture these unique outcomes. Example quotes from the focus groups are provided for each of the core areas; not that this is not an exhaustive list, but rather a selection intended to illustrate the sources of the outcomes.

## Supplemental 7. Mean Delphi survey outcome ratings.

|  | Delphi 1 | | Delphi 2 | | Delphi 3 | |
| --- | --- | --- | --- | --- | --- | --- |
|  | Patient & Caregiver  Mean Ratings (N) | Health Professional  Mean Ratings (N) | Patient & Caregiver  Mean Ratings (N) | Health Professional  Mean Ratings (N) | Patient & Caregiver  Mean Ratings (N) | Health Professional  Mean Ratings (N) |
| Adaptive Functioning | 8 (90) | 8 (30) | 8 (83) | 8 (26) | 8 (63) | 8 (21) |
| Daily Living Skills | 8 (89) | 7 (29) | 8 (81) | 8 (27) | 8 (62) | 8 (21) |
| Cognitive Functioning | 9 (90) | 8 (30) | 9 (82) | 9 (27) | 9 (63) | 9 (21) |
| Emotional  Dysregulation | 7 (90) | 6 (30) | 7 (83) | 6 (27) | 7 (63) | 6 (21) |
| MRS Brain Creatine | 9 (87) | 8 (27) | 9 (79) | 8 (27) | 8 (62) | 8 (21) |
| Seizures/  Convulsions | 8 (89) | 8 (29) | 8 (79) | 9 (27) | 8 (61) | 9 (21) |
| Serum/Plasma Guanidinoacetate (GAA) | 7 (77) | 8 (24) | 7 (77) | 8 (25) | 7 (58) | 8 (20) |
| Expressive  Communication | 8 (90) | 8 (30) | 8 (82) | 8 (27) | 8 (63) | 8 (21) |
| Fine Motor Functions | 8 (87) | 6 (29) | 8 (79) | 7 (27) | 8 (60) | 7 (21) |
| MRI Brain, General | 8 (84) | 8 (28) | 8 (79) | 8 (27) | 8 (62) | 8 (21) |
| MRS Brain  Guanidinoacetate | 8 (84) | 8 (23) | 8 (77) | 8 (25) | 8 (62) | 8 (19) |
| Serum/Plasma Creatine | 8 (85) | 7 (24) | 7 (80) | 8 (27) | 8 (60) | 8 (21) |
| Aggressive Behaviors | 7 (90) | 7 (30) | 7 (83) | 7 (27) | 7 (63) | 7 (21) |
| Caregiver Burden | 7 (88) | 7 (27) | 7 (79) | 8 (26) | 7 (60) | 8 (19) |
| EEG Epileptic Potentials | 8 (87) | 7 (28) | 8 (80) | 8 (27) | 8 (63) | 7 (21) |
| Executive Functioning | 8 (90) | 8 (30) | 8 (83) | 8 (26) | 8 (63) | 7 (20) |
| Independence | 8 (89) | 7 (29) | 8 (80) | 7 (27) | 8 (62) | 7 (21) |
| Developmental Delay | 8 (89) | 8 (30) | 8 (82) | 9 (27) | 9 (63) | 9 (21) |
| Intellectual Disability | 8 (90) | 8 (30) | 9 (83) | 8 (27) | 9 (61) | 9 (21) |
| Life Expectancy | 7 (84) | 7 (26) | 7 (78) | 7 (27) | 7 (59) | 6 (20) |
| MRS Brain  Phosphocreatine | 8 (86) | 8 (22) | 8 (79) | 8 (26) | 8 (61) | 9 (20) |
| Receptive Language | 8 (90) | 8 (30) | 8 (83) | 8 (27) | 8 (63) | 8 (21) |
| Treatment Access | 8 (87) | 7 (27) | 8 (80) | 8 (25) | 8 (61) | 8 (18) |
| Treatment Efficacy | 9 (88) | 8 (28) | 9 (80) | 9 (28) | 9 (61) | 9 (20) |
| Treatment Practicability | 8 (88) | 7 (29) | 8 (80) | 8 (27) | 8 (61) | 8 (20) |
| Treatment Safety | 9 (88) | 8 (29) | 9 (79) | 9 (27) | 9 (61) | 9 (20) |
| Treatment Tolerability | 8 (88) | 8 (29) | 8 (80) | 8 (27) | 8 (61) | 9 (20) |
| Coordination | 7 (87) | 6 (29) | 7 (79) | 7 (27) | 7 (60) | 7 (20) |
| EEG Abnormal Unspecific | 8 (88) | 6 (28) | 8 (80) | 6 (27) | 8 (62) | 7 (21) |
| Healthcare System  Resource Availability | 7 (86) | 7 (27) | 7 (79) | 7 (26) | 7 (60) | 7 (19) |
| Low Muscle Tone,  Hypotonia | 7 (86) | 7 (29) | 7 (79) | 7 (27) | 7 (60) | 7 (20) |
| MRI Brain  Basal Ganglia | 8 (82) | 7 (27) | 7 (77) | 7 (27) | 7 (61) | 7 (20) |
| MRI Brain Atrophy | 8 (83) | 7 (29) | 8 (79) | 7 (27) | 7 (62) | 6 (21) |
| MRI Brain White Matter | 8 (83) | 7 (28) | 8 (79) | 7 (27) | 8 (61) | 7 (21) |
| MRI Cerebellar &  Brainstem | 8 (83) | 7 (28) | 8 (77) | 7 (27) | 7 (61) | 6 (20) |
| Self-Protection | 7 (89) | 5 (29) | 7 (81) | 5 (27) | 7 (62) | 5 (21) |
| Sensory Processing  Issues | 7 (89) | 6 (30) | 7 (82) | 6 (27) | 7 (63) | 6 (21) |
| Urine Creatine/Creatinine | 8 (88) | 7 (25) | 8 (81) | 8 (28) | 7 (62) | 8 (21) |
| Walking Ability | 7 (86) | 7 (27) | 7 (79) | 7 (27) | 6 (60) | 7 (21) |
| Ataxia | 7 (84) | 7 (28) | 7 (77) | 7 (26) | 7 (59) | 7 (20) |
| Autism Spectrum  Disorder (ASD) | 7 (89) | 7 (30) | 7 (81) | 8 (27) | 6 (63) | 7 (21) |
| QTc and Prolonged QTc | 7 (88) | 6 (27) | 7 (79) | 6 (27) | 7 (62) | 6 (21) |
| Attention Deficit/  Hyperactivity Disorder (ADHD) | 7 (89) | 7 (30) | 7 (82) | 6 (26) | 7 (63) | 6 (20) |
| CSF Creatine | 7 (80) | 6 (22) | 7 (77) | 6 (27) | 7 (58) | 6 (19) |
| CSF Guanidinoacetate  (GAA) | 7 (74) | 6 (22) | 6 (71) | 6 (25) | 6 (55) | 5 (18) |
| Healthcare System  Resource Use | 7 (87) | 7 (28) | 7 (78) | 7 (26) | 7 (60) | 7 (19) |
| High Muscle Tone,  Spasticity | 6 (84) | 7 (28) | 6 (77) | 7 (27) | 6 (60) | 7 (20) |
| Sleep Disturbance | 6 (85) | 7 (28) | 6 (79) | 7 (27) | 6 (59) | 7 (20) |
| Social Relationships | 7 (89) | 6 (28) | 7 (81) | 6 (27) | 7 (62) | 6 (21) |
| Urine Guanidinoacetate (GAA) | 7 (80) | 7 (24) | 7 (74) | 7 (26) | 6 (60) | 6 (20) |
| Academic Achievement | 6 (90) | 6 (30) | 6 (82) | 5 (27) | - | - |
| Anxiety | 6 (90) | 6 (30) | 6 (82) | 6 (27) | - | - |
| Arrhythmia | 6 (89) | 6 (29) | 6 (81) | 6 (27) | - | - |
| Bladder Incontinence | 6 (87) | 5 (27) | 6 (81) | 5 (26) | - | - |
| Body Length | 5 (88) | 5 (29) | 5 (80) | 5 (27) | - | - |
| Breath-Holding | 6 (85) | 5 (26) | 6 (79) | 5 (26) | - | - |
| Cardiac Events | 7 (88) | 6 (29) | 7 (80) | 6 (27) | - | - |
| Cardiomyopathy | 6 (88) | 5 (28) | 6 (81) | 6 (27) | - | - |
| Constipation | 6 (88) | 6 (30) | 5 (83) | 5 (27) | - | - |
| Depression | 5 (88) | 6 (30) | 5 (82) | 6 (27) | - | - |
| Dystonia | 6 (85) | 7 (29) | 6 (78) | 7 (27) | - | - |
| Educational Resource Use | 7 (86) | 7 (28) | 7 (79) | 7 (26) | - | - |
| Failure to Thrive | 7 (86) | 6 (28) | 6 (80) | 6 (27) | - | - |
| Feeding and Eating  Problems | 6 (88) | 6 (30) | 6 (83) | 6 (27) | - | - |
| Frustration | 7 (90) | 5 (30) | 6 (83) | 5 (27) | - | - |
| Intolerance to Change | 6 (90) | 6 (30) | 6 (83) | 6 (27) | - | - |
| Low Energy | 6 (85) | 5 (29) | 6 (79) | 5 (27) | - | - |
| Muscle Weakness | 7 (86) | 6 (27) | 6 (78) | 6 (27) | - | - |
| Obsessive Compulsive Behavior | 6 (90) | 6 (30) | 6 (83) | 6 (27) | - | - |
| Oppositional Defiant  Behavior | 7 (88) | 6 (30) | 6 (82) | 6 (27) | - | - |
| Pain | 6 (89) | 6 (30) | 6 (82) | 6 (27) | - | - |
| Physical Activity | 7 (87) | 5 (29) | 6 (80) | 5 (27) | - | - |
| Physical Endurance | 6 (86) | 5 (29) | 6 (79) | 5 (27) | - | - |
| Psychosis | 5 (85) | 6 (30) | 5 (81) | 6 (27) | - | - |
| Regression | 7 (90) | 7 (30) | 7 (82) | 7 (27) | - | - |
| Repetitive Behaviors | 7 (90) | 6 (30) | 6 (83) | 6 (27) | - | - |
| Restlessness/  Agitation | 7 (90) | 6 (30) | 7 (83) | 6 (27) | - | - |
| Self-Injury | 6 (88) | 7 (30) | 6 (82) | 7 (27) | - | - |
| Vision Impairment | 5 (88) | 6 (30) | 5 (82) | 5 (27) | - | - |
| Vomiting | 5 (87) | 5 (30) | 4 (82) | 5 (27) | - | - |
| Weight | 7 (88) | 6 (29) | 6 (80) | 6 (27) | - | - |
| Diarrhea | 5 (86) | 4 (30) | - | - | - | - |
| Gastroesophageal Reflux Disease (GERD) | 5 (87) | 5 (30) | - | - | - | - |
| Hearing Impairment | 5 (90) | 6 (30) | - | - | - | - |
| Strabismus and  Gaze Palsy | 5 (84) | 5 (30) | - | - | - | - |

Note: Mean ratings for each candidate outcome during the three Delphi survey rounds.

##

## Supplemental 8. Consensus workshop voting results for the candidate outcomes.

| **Outcome** | **1-Definitely In**  **n (%)** | **2-Maybe**  **n (%)** | **3-Definitely Out**  **n (%)** |
| --- | --- | --- | --- |
| Seizure/Convulsions | 24 (69) | 11 (31) | 0 (0) |
| Caregiver Burden | 2 (6) | 19 (54) | 14 (40) |
| Emotional Dysregulation | 21 (60) | 14 (40) | 0 (0) |
| Aggressive Behaviors | 1 (3) | 12 (34) | 22 (63) |
| Cognitive Functioning | 23 (66) | 12 (34) | 0 (0) |
| Intellectual & Developmental Disability | 5 (14) | 13 (37) | 17 (49) |
| Adaptive Functioning | 30 (86) | 5 (14) | 0 (0) |
| Expressive Language | 3 (9) | 2 (77) | 5 (14) |
| Receptive Language | 0 (0) | 22 (63) | 13 (37) |
| Independence | 1 (3) | 19 (54) | 15 (43) |
| Executive Functioning | 1 (3) | 14 (40) | 20 (57) |
| Fine Motor Functions | 0 (0) | 14 (40) | 21 (60) |
| MRS Brain Creatine | 29 (83) | 6 (17) | 0 (0) |
| MRS Brain Phosphocreatine | 2 (6) | 15 (43) | 18 (51) |
| MRI Brain General | 2 (6) | 15 (43) | 18 (51) |
| MRS Brain Guanidinoacetate | 0 (0) | 19 (54) | 16 (46) |
| EEG Epileptic Potentials | 0 (0) | 13 (37) | 22 (63) |
| Serum Plasma Creatine | 5 (14) | 10 (29) | 20 (57) |
| Serum Plasma Guanidinoacetate | 14 (40) | 8 (23) | 13 (37) |
| Life Expectancy | 0 (0) | 6 (17) | 29 (83) |

Note: There were 25 total voting consensus workshop participants. However, there were 35 voting responses as some health professionals voted twice, once for CTD and once for GAMT.
